# Spatial transcriptomics reveals segregation of tumor cell states in glioblastoma and marked immunosuppression within the perinecrotic niche

**DOI:** 10.1186/s40478-024-01769-0

**Published:** 2024-04-22

**Authors:** Mengyi Liu, Zhicheng Ji, Vaibhav Jain, Vanessa L. Smith, Emily Hocke, Anoop P. Patel, Roger E. McLendon, David M. Ashley, Simon G. Gregory, Giselle Y. López

**Affiliations:** 1 Computational Biology and Bioinformatics Program, Duke University School of Medicine, Durham, NC 27710 USA; 2 The Preston Robert Tisch Brain Tumor Center, Duke University School of Medicine, Durham, NC 27710 USA; 3 Duke Molecular Physiology Institute, Duke University School of Medicine, Durham, NC 27705 USA; 4 Department of Biostatistics and Bioinformatics, Duke University School of Medicine, Durham, NC 27710 USA; 5 Department of Neurosurgery, Duke University School of Medicine, Durham, NC 27710 USA; 6 Department of Pathology, Duke University School of Medicine, Durham, NC 27710 USA

**Keywords:** Glioblastoma, Spatial transcriptomics, Single-cell sequencing, Tumor microenvironment, Perinecrotic niche, Perivascular niche

## Abstract

Glioblastoma (GBM) remains an untreatable malignant tumor with poor patient outcomes, characterized by palisading necrosis and microvascular proliferation. While single-cell technology made it possible to characterize different lineage of glioma cells into neural progenitor-like (NPC-like), oligodendrocyte-progenitor-like (OPC-like), astrocyte-like (AC-like) and mesenchymal like (MES-like) states, it does not capture the spatial localization of these tumor cell states. Spatial transcriptomics empowers the study of the spatial organization of different cell types and tumor cell states and allows for the selection of regions of interest to investigate region-specific and cell-type-specific pathways. Here, we obtained paired 10x Chromium single-nuclei RNA-sequencing (snRNA-seq) and 10x Visium spatial transcriptomics data from three GBM patients to interrogate the GBM microenvironment. Integration of the snRNA-seq and spatial transcriptomics data reveals patterns of segregation of tumor cell states. For instance, OPC-like tumor and NPC-like tumor significantly segregate in two of the three samples. Our differentially expressed gene and pathway analyses uncovered significant pathways in functionally relevant niches. Specifically, perinecrotic regions were more immunosuppressive than the endogenous GBM microenvironment, and perivascular regions were more pro-inflammatory. Our gradient analysis suggests that OPC-like tumor cells tend to reside in areas closer to the tumor vasculature compared to tumor necrosis, which may reflect increased oxygen requirements for OPC-like cells. In summary, we characterized the localization of cell types and tumor cell states, the gene expression patterns, and pathways in different niches within the GBM microenvironment. Our results provide further evidence of the segregation of tumor cell states and highlight the immunosuppressive nature of the necrotic and perinecrotic niches in GBM.

**Supplementary Information:**

The online version contains supplementary material available at 10.1186/s40478-024-01769-0.

## Introduction

Glioblastoma (GBM), the most common primary malignant brain tumor in adults, remains almost universally lethal, with a median survival of less than two years [24]. Single-cell technologies have been successful in characterizing cell types in the tumor microenvironment (TME) of GBMs at a molecular level [17, 25], which is critical for understanding their pathogenicity. Previous studies characterized the tumor cells in GBM into four main cell states, aligning with neural development in brain: (i) neural progenitor-like (NPC-like), (ii) oligodendrocyte-progenitor-like (OPC-like), (iii) astrocyte-like (AC-like) and (iv) mesenchymal like (MES-like) states [17]. Although single-cell approaches have facilitated the identification of these cell states, the inherent requirement for tissue dissociation means that this technology cannot spatially localize the tumor cells within the tissue, nor can it inform how tumor cells in different cell states interact with each other.

Spatial transcriptomic technologies, which capture gene expression in the context of tissue structure [2, 14], offer a new approach to studying the TME. Spatial transcriptomics enables the interrogation of histologic hallmarks associated with specific gene expression patterns within the tissue. Two hallmarks of GBM are the characteristic niches of palisading necrosis and microvascular proliferation [9]. Spatial transcriptomic characterization of GBM will enable the regional selection of these niches and allow for the interrogation of the specific pathways, akin to other cancer types in which the presence of distinct perivascular macrophage populations correlates with increased tumor angiogenesis, poor prognosis, and recurrence after chemotherapy [13].

Previous studies have integrated public single-cell RNA-sequencing (scRNA-seq) with their spatial transcriptomics data. These studies defined “modules” according to spatial transcriptional programs and confirmed the spatial segregation of these modules [22], rather than using cell-state categories [17]. A second study defined five spatially distinct transcriptional programs to establish that these subgroups are spatially segregated and engage in different functions within GBM [21]. Both studies lay the foundation for interrogating the GBM TME with the integration of single-cell and spatial transcriptomics technologies. However, the question of how hallmark histopathologic characteristics of GBM (microvascular proliferation, perivascular niche, necrosis) correlate to transcriptional programs and cell states remains unknown. In this study, we used paired snRNA-seq and spatial transcriptomics to interrogate the question of how macrostructural features of GBM correlate to tumor cell states. We uncovered segregation patterns between different tumor cell states, identified an OPC-enriched niche within the perivascular space, and identified marked immunosuppression within the perinecrotic niche.

## Materials and methods

### Tissue preparation

SnRNA-seq (Chromium) and spatial transcriptomics (Visium) experiments were carried out in frozen GBM tissue from three patients who had consented to donate excess tissue from their tumors to be banked by the Duke Brain Tumor Biorepository. Approval for the following studies using this de-identified tissue was obtained from the Institutional Review Board of Duke University (Pro00105756).

### Library preparation for snRNA-seq

Nuclei isolation was performed using the 10x Genomics Nuclei Isolation kit (10x Genomics, Pleasanton, CA, USA). Briefly, 5-30 mg of tissue was collected from 50 µm sections from each GBM block. Nuclei were isolated using lysis buffer in conjunction with manual tissue homogenization. Crude nuclear pellets were washed and passed through a Debris Removal Buffer. Nuclear pellets were then washed and counted on a Nexcelom Cellometer K2 (Nexcelom Biosciences, Lawrence, MA, USA) and titrated. To generate single nuclei libraries using the Chromium 3’ v3.1 Gene Expression assay (10x Genomics, Pleasanton, CA, USA), nuclei suspensions were loaded on the 10x Genomics Chromium Controller Single-Cell Instrument (10x Genomics, Pleasanton, CA, USA) where they were combined with reverse transcription reagents, gel beads, and oil to generate single-nuclei gel bead in emulsions (GEMs). GEM-Reverse Transcription (GEM-RT) was performed in an Eppendorf Mastercycler Pro (cat#90,030,020, Eppendorf): 53 °C for 45 min, 85 °C for 5 min; held at 4 °C. After RT, GEMs were broken and the single-strand cDNA was purified with DynaBeads MyOne Silane Beads (cat#37002D, Thermo Fisher Scientific). cDNA was amplified (Eppendorf Mastercycler Pro, cat#950,030,020, Eppendorf): 98 °C for 3 min; cycled 11–13 × : 98 °C for 15 s, 67 °C for 20 s, and 72 °C for 1 min; 72 °C for 1 min; held at 4 °C. Amplified cDNA product was purified with the SPRIselect Reagent Kit (0.6 × SPRI) (cat#B23318, Beckman Coulter). Indexed sequencing libraries were constructed using the reagents in the Chromium Single-Cell 3′ v3.1 Library Kit by (1) fragmentation, end repair and A-tailing; (2) SPRIselect cleanup; (3) adapter ligation; (4) post ligation cleanup with SPRIselect; (5) sample index PCR; and (6) PostindexPCR cleanup. The barcoded sequencing libraries were quantified by quantitative PCR (cat#KK4824, KAPA Biosystems Library Quantification Kit for Illumina platforms). Sequencing of the libraries was carried out by Azenta (South Plainfield, NJ).

### snRNA-seq data processing

We processed each of the snRNA-seq dataset separately with the procedure below:

Raw FASTQ files were processed with 10x Genomics Cell Ranger 7.0.0 [30] to align sequencing reads in FASTQ files to the hg38 human reference transcriptome and to generate outputs for single cell analysis. R package Seurat 4.4.0 [10] was used for the data processing including quality control, normalization, scaling, dimensionality reduction and clustering. Three filtering steps were used to filter out low quality cells: (1) SoupX [27] to remove potential ambient RNA; (2) cells expressing fewer than 500 genes or with more than 5% reads aligned to mitochondrial genes were removed using Seurat; (3) after the clustering step, DoubletFinder [15] was used to remove potential doublets in the droplet-based sequencing method.

### Cell type and cell state annotation for snRNA-seq

**Fig. 1:**
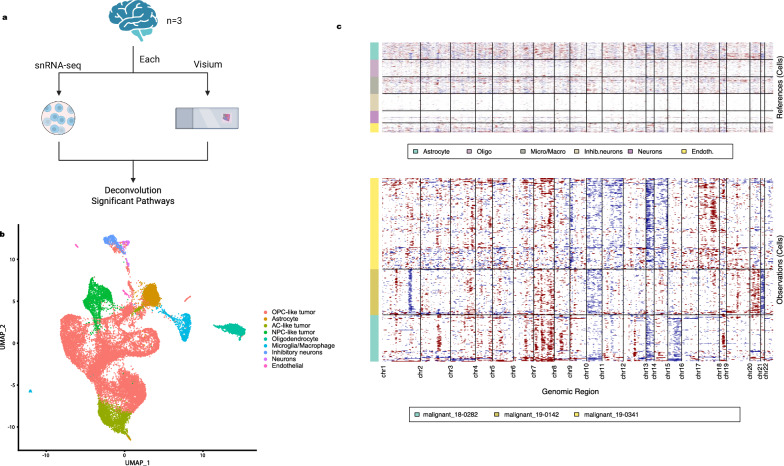
**a** Workflow of the study. From each GBM brain, both snRNA-seq and Visium spatial transcriptomics data were generated as paired samples. The single nuclei data and spatial data were processed separately, then integrated for downstream analysis. **b** Combined snRNA-seq data from three GBMs with annotation of cell types and tumor cell states. **c** Combined inferCNV results from three GBMs. Chromosome 7 gain and 10 loss was observed in all samples, and each sample had distinct copy number variations (CNV), demonstrating heterogeneity in CNV

For each sample, the assignment of cell types to each cluster was guided by canonical marker gene expression (Fig. 1, [19]). For annotation of different tumor cell states, gene lists for MES-like, AC-like, OPC-like, and NPC-like states were referenced from Neftel et al. [17]. Cell state lineage scores of each tumor cell were calculated as the mean expression from genes of a particular cell state divided by the mean expression of all genes in that cell. A mean lineage score for each cell state was calculated for each cluster, and the cell state with the highest lineage score was assigned as the cluster cell state. Here, we have used an approximation when assigning each cluster as one cell state, and we did not discuss transitional cell states in this paper for simplicity.

### Integration of snRNA-seq data for visualization

We performed integration of the samples with Seurat’s anchor-based integration [5], which corrects for batch effects (see Additional file 1: Fig. S1). Before the clustering step, we used the R package clustree [29] to choose the optimal resolution to use (Additional file 2: Fig. S2). A resolution of 0.5 was used for FindClusters() in Seurat to produce clustering results by the manifold learning technique UMAP (Uniform Manifold Approximation and Projection) [16].

### Copy number variation analysis

The copy number content for tumor and normal populations was confirmed using InferCNV [11], with parameter denoise being set to TRUE in the run() function.

### Chromosomal microarray

To confirm the copy number alterations identified by InferCNV in the single nucleus RNA sequencing data, we performed chromosomal microarray on tissue from the same case, but from different blocks which were available at the time of the study. Chromosomal microarray analysis was performed using the ThermoFisher Oncoscan array. DNA was isolated from fresh frozen tissue from each case using the Qiagen DNeasy Blood and Tissue Kit (Catalog Number 65904). Following DNA isolation, molecular inversion probes were annealed to the DNA, ligated, cleaved, amplified, fragmented, and hybridized to the array. Washed arrays (Affymetrix Fluidics Station 450) were scanned (Affymetrix GeneChip Scanner 3000) and analyzed using the Affymetrix ChAS software. Copy number variants were evaluated based on professional organization and World Health Organization guidance [3, 18] and reported based on the GRCh37/hg19 genome build.

### Tissue and library preparation for spatial transcriptomics

Tissue embedded in optimal cutting temperature (OCT) compound was cut to 10 μm and placed on a Tissue Optimization Slide (TO Slide) to determine permeabilization conditions. Once optimal conditions were determined (12 min permeabilization), new tissue slices were placed within a 42 mm^2^ field on the Visium expression slide containing 5000 barcoded probes (10x Genomics, Pleasanton, CA). Tissue was fixed and stained with Hematoxylin and Eosin (H&E) then permeabilized to release mRNA, which binds to spatially barcoded capture probes, allowing for the capture of gene expression information. Barcoded cDNA was synthesized from the slide surface from captured mRNA, denatured and cleaved, and transferred for cDNA amplification and standard NGS library preparation. Briefly, the barcoded, amplified cDNA was enzymatically fragmented, purified and size selected. Adapters were then ligated to each fragment followed by a sample index PCR. The libraries were sequenced to an average of 50,000 reads/probe on a paired end, dual indexed flow cell in the format of 28 × 10 × 10 × 90.

### Visium data analysis

Image processing aligned the slide's barcoded spot pattern to the H&E input slide image to discriminate tissue and background in the slide image. After the automated alignment and tissue identification processes, a full resolution image was used as input in order to prepare data for visualization within 10x Genomics Loupe Browser 4.1.0 [26]. The 10x Genomics Space Ranger 1.2.1 was used to process RNA-seq output and bright field microscope images to align reads, generate feature-spot matrices, perform clustering and gene expression analysis, and place spots in spatial context on the slide image. Space Ranger includes two pipelines relevant to spatial gene expression experiments in which the program mkfastq wraps Illumina’s bcl2fastq to demultiplex the sequencing runs and to convert barcode and read data to FASTQ files. Space Ranger count takes a bright field slide image and FASTQ files from mkfastq for alignment, tissue detection, fiducial detection, and barcode/UMI counting. The pipeline uses the spatial barcodes to generate feature-spot matrices, determine clusters and perform gene expression analysis. These pipelines combine Visium-specific algorithms with the RNA-seq STAR [7] aligner. Output was delivered in standard visualization formats that are augmented with spatial information.

Secondary statistical analysis was based on the R package Seurat 4.4.0 [10] to perform quality control and subsequent analyses on the spot-level expression data. Minimal filtering was done to keep the majority of spots for downstream analysis (in sample 18-0282, spots with over 5% reads aligned to mitochondrial genes were filtered out). For normalization and variance stabilization of molecular counts, we used the function SCTransform().

### Niche selection

Perivascular niche and generic tumor regions were selected by reviewing H&E images in the 10x Genomics Loupe browser by a board-certified neuropathologist (GYL). The palisading necrosis niche was selected based on the spots with normalized VEGFA expression values larger than 3. VEGFA was chosen rather than just histologic evaluation alone for identification of palisades around necrosis because it captured both histologically identifiable palisading necrosis as well as areas of increased cellular density without necrosis that likely represented palisades where the necrosis was outside the plane of section. Overlapping spots between the palisading necrosis, perivascular niche and generic tumor region were removed before downstream analysis.

### Integration of snRNA-seq with spatial transcriptomics data

The R package RCTD [4] was used for deconvolution of the snRNA-seq and spatial data. The matched and annotated snRNA-seq data for each spatial transcriptomics sample was used as the reference for deconvolution. The snRNA-seq was performed on the same block that the spatial transcriptomics was performed on. Since RCTD needs a minimum of 25 cells for each cell type in the reference data, cell types with fewer than 25 cells were removed from the single cell reference.

### Statistical test for cell type localization

We used the function cor.test() in the R stats package [20] to calculate the Pearson correlation coefficient between the deconvoluted cell type proportions of each cell type pair, as well as the *p* values for the correlation. Adjusted *p* values were then calculated using the Benjamini–Hochberg procedure [5].

### Differentially expressed genes (DEGs) analysis

We performed DEGs analysis for both of the following: not adjusting for cell type proportions, and with adjustment for cell type proportions. Note that the two analyses differ by the design matrix described below. The R package limma [23] was used to identify DEGs between regions of interest in spatial transcriptomic spots. In the analysis with adjustment of cell type proportions, we added the deconvolution result in the design matrix such that the different tumor cell states were combined as “tumor” in one column, and the non-tumor cell types has their own columns in the design matrix. This allows us to adjust for effects of preferential localization of tumor versus non-tumor cells. The function topTable() was used to find the DEGs, and again, Benjamini–Hochberg procedure [5] was used to adjust the *p* values. The significant cutoff for adjusted *p* values was set to 0.05.

### Pathway analyses

Gene Ontology (GO) Enrichment Analysis [8] was performed using the top 100 differentially expressed genes with the highest absolute values of log fold change (logFC) in both positive and negative directions between the niches of interest and the generic tumor. We chose the “Biological process” and “Homo sapiens” option. FDR < 0.05 was used to identify significantly enriched GO terms.

Ingenuity Pathway Analysis (IPA, version 01-20-04): We identified significant pathways comparing niches of interest and the generic tumor region, then performed comparison across samples to find pathways consistently altered between samples. *p* value < 0.05 and FDR < 0.05 were the cutoffs. No cutoffs were used for the Expression Log Ratios. Under the “General Settings”, population of genes to consider for *p* value calculations: reference set was “Ingenuity Knowledge Base” (genes only), assessing both direct and indirect relationships. Default settings were used for the “Networks Filter” and the “Mutation Filter”. We used all node types, and all data sources (default). For “miRNA Confidence Filter”, we used “Experimentally Observed and High” (predicted). Species was restricted to Human, with the “Stringent” filter option. For the “Tissues and Cell Lines Filter”, we included astrocytes, all endothelial cells, immune cells, neurons, vascular smooth muscle cells, nervous system cells, CNS cell lines, and immune cell lines.

### Gradient analysis

The annotation of gradient of regions, by a board-certified neuropathologist (GYL, see Additional file 3: Fig. S3), is different from the niche annotation since it breaks down the spatial regions into smaller segments and offers a more fine-grained layer of annotation. The R package limma [23] was used to find the DEGs in each region compared to the rest of the regions (same strategy as in DEGs analysis). Like the DEGs analysis, we also performed gradient analysis for both: not adjusting for cell type proportions, and with adjustment of cell type proportions.

To plot the heatmaps, the gene expression of each region was scaled to have a mean of 0 and a standard deviation of 1. For each region, up to the top 10 genes with the highest logFC (positive enrichment in that region, adjusted *p* value < 0.05) were plotted. Ribosomal genes were excluded from the analysis. For the cell type localization heatmap, we again scaled the deconvolution results to have a mean of 0 and a standard deviation of 1 before plotting the cell types across the gradient of regions. In IPA, we used FDR cutoff of 0.05 and *p* value of 0.05. As part of the requirement for running IPA, we also used logFC cutoff (typically − 0.1 to 0.1) to get the number of genes analyzed down to 5000.

## Results

### Overview of the workflow of study

We obtained paired snRNA-seq (Chromium) and spatial transcriptomics (Visium) data from three human GBM samples, along with related clinical data (Additional file 18: Table S1). All samples had molecular features of glioblastoma, including TERT promoter mutation (C228T), lack of IDH1 R132H mutation (confirmed by immunohistochemistry in all cases, and additionally by sequencing in two cases), and gain of chromosome 7 (Additional file 18: Table S1). The single nuclei data and spatial data were processed separately, then integrated to identify cell type localization and pathways in regions of interest (Fig. 1a).

### Single nucleus RNA-seq data reveals tumor cells in different cell states

We first processed the snRNA-seq data from each of the three GBM samples to identify tumor and non-tumor cell types (Fig. 1b) with marker genes [19] (see Methods) and to study chromosomal copy number variations. All three GBM samples demonstrated chromosome 7 gain and chromosome 10 loss features (Fig. 1c), which are characteristic of canonical GBMs [6]. We observed different patterns of additional chromosomal alterations in each individual sample: sample 18-0282 showed additional loss of chromosome 13 and 15, sample 19-0142 demonstrated chromosome 21 loss, and sample 19-0341 showed chromosome 13 loss. These findings were validated by performing chromosomal microarray on separate blocks from each of the cases, which confirmed the presence of chromosome 7 gain and chromosome 10 loss in each case. For each case, while chromosome 7 gain and chromosome 10 loss remained consistent between inferCNV and chromosomal microarray, there were different additional genetic alterations identified by chromosomal microarray (Additional file 4: Fig. S4, Additional file 19: Table S2). This likely reflects a combination of decreased sensitivity of inferCNV compared to chromosomal microarray, as well as intratumoral heterogeneity, as different blocks from the same tumors were used for the chromosomal microarray than were used for spatial transcriptomics. Importantly, the clonal driver mutations (7 gain/10 loss) were constant across all cases and all modalities.

To identify different cell states of tumor within the samples (MES-like, NPC-like, AC-like and OPC-like states), we used the gene lists for each cell state from Neftel et al. [17] (see Methods, Additional file 20: Table S3). OPC-like and NPC-like tumor cells were found in all three samples, while AC-like tumor cells were found in two of the samples (19-0142 and 19-0341, see Additional file 5: Fig. S5). The greatest number of cell types were identified within sample 19-0341, including non-tumor populations such as inhibitory neurons and excitatory neurons, likely because this sample represents infiltrative tumor edge, whereas the other two samples represent tumor core.

### Spatial transcriptomics data pinpoints the localization of different tumor cell states

**Fig. 2:**
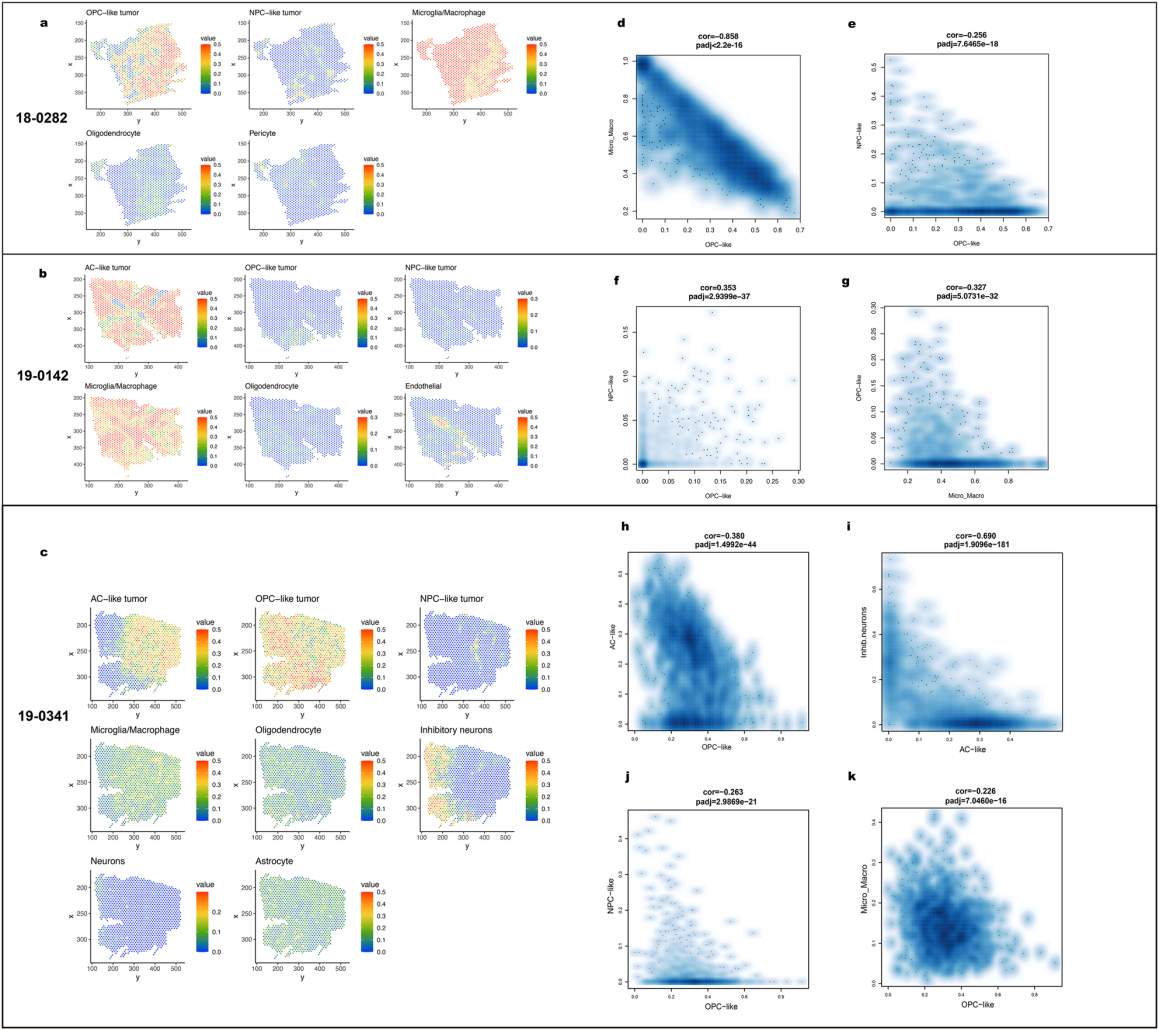
**a**–**c** Deconvolution results from RCTD for samples 18-0282, 19-0142, and 19-0341, respectively, showing localization of each cell type. “Neuron” refers to cells expressing both excitatory and inhibitory neuron markers. **d**–**k** Correlation between cell type pairs, calculated with cor.test() in R. Complete correlation plots in Additional file 6: Fig. S6

To understand how different cell types and tumor cell states localize within specific regions of the tissue, we integrated the snRNA-seq data for each sample with the matched spatial transcriptomics data to perform cell type deconvolution using RCTD [4]. This process allows us to identify where tumor cells of differing cell states (AC-like, NPC-like, OPC-like and MES-like) are located spatially, as well as the location of non-tumor cells on the slide. Our deconvolution results (Fig. 2a-c) reveal that samples 18-0282 and 19-0142 are both dominated by a single tumor cell state (OPC-like tumor and AC-like tumor, respectively), while 19-0341 demonstrates high abundance of both OPC-like and AC-like tumor (OPC-like is higher). To validate these findings, we compared to the snRNA-seq data from the same block, which showed similar fractions of the tumor cell states. In the single nucleus data, the majority of 18-0282 is OPC-like tumor; in 19-0142, AC-like tumor consists of an overwhelmingly dominating amount; in 19-0341, OPC-like tumor makes up the majority, while there is also AC-like and NPC-like tumor (Additional file 5: Fig. S5).

We evaluated whether tumor cells of different states were intermingled or tended to segregate from each other to form niches for a given cell state. Our studies found that, in most instances, different tumor cell states tended to segregate to distinct locations (Fig. 2a-c). For example, within sample 19-0341, AC-like tumor, OPC-like tumor, and NPC-like tumor each localized to distinct spatial regions within the tumor, and did not appear to overlap to any significant extent (Fig. 2c).

While this pattern seemed apparent on visual inspection of the deconvolution results, we performed correlation tests for each pair of cell states and cell types to statistically assess for both segregation and co-localization patterns. For 19-0341, the segregation between AC-like tumor and OPC-like tumor is significant (adjusted *p* value = 1.50e − 44, Fig. 2h); AC-like tumor and inhibitory neurons also segregate significantly (adjusted *p* value = 1.91e − 181, Fig. 2i). Although OPC-like tumor and NPC-like tumor are present in all three samples, the localization patterns between these two cell states demonstrated variability between tumors (Fig. 2e, f, j). In samples 18-0282 and 19-0341, OPC-like tumor and NPC-like tumor significantly segregate (adjusted *p* values = 7.65e − 18, 2.99e − 21); however, in 19-0142, the two cell states significantly co-localize (adjusted *p* values = 2.94e − 37). 19–0142 comprises nearly entirely AC-like tumor cells; in this situation, it may be that the very low numbers of NPC-like and OPC-like cells form a common niche; alternatively, it is possible that the RCTD is less accurate when the number of cells for a given state are very low. Interestingly, among the three samples, the most consistent cell-type pair localization patterns were segregation between microglia/macrophage and OPC-like tumors, and between microglia/macrophage and oligodendrocytes (Fig. 2g, k, Additional file 6: Fig. S6).

### Differentially expressed genes (DEG) and significant pathways uncovered in the spatial niches of interest

Next, we set out to identify DEGs and pathways in the regions of interest, namely, the palisading necrosis niche and the perivascular niche. We selected spots with high VEGFA expression (see Methods) as the palisading necrosis niche. A board-certified neuropathologist identified the perivascular niche and the generic tumor region (tumor away from both niches) by aligning spots with the morphological features of the H&E images (Additional files 7, 8, 9, 10: Figs. S7, S8, S9, S10).

We then performed DEGs analysis comparing each of the niches and the generic tumor region. As we observed in Fig. 2, different regions of the tissue are enriched for different cell types and thus DEGs between regions are related to cell classes (e.g., when tumor cells tend to highly express certain genes in a region). To adjust for the effects due to cell types, we incorporated the deconvolution results in the DEGs analysis (see Methods).

Two of the cases (18-0282 and 19-0142), histologically representing tumor core, had high tumor density and contained regions of microvascular proliferation and palisading necrosis. The third sample (19-0341) came from the infiltrative tumor edge, demonstrated lower tumor density and lacked microvascular proliferation and palisading necrosis within the sampled region. Thus, we focused on the first two samples when evaluating the niches of interest moving forward.

**Fig. 3:**
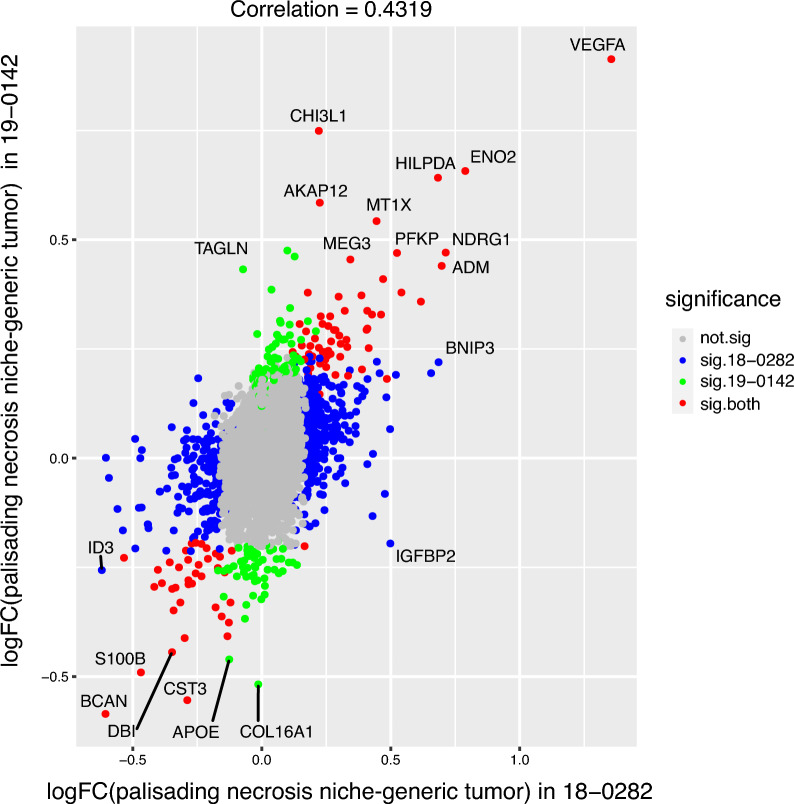
Plot for samples 18-0282 and 19-0142, logFC of gene expression in palisading necrosis niche compared to the generic tumor region, cell type adjusted. Whether the logFC of the gene is significant is shown in the legend. Adjusted *p* values < 0.05 were labeled as significant

To evaluate the similarity between the DEGs in the niches of interest between the two samples, we calculated the correlation between the logFC values of gene expression in each niche compared to the generic tumor region. In the case of DEGs analysis with adjustment of cell types, we found a positive correlation of 0.43 in the palisading necrosis niche between the two samples (Fig. 3). Several genes are significantly upregulated in the palisading necrosis niche with logFC higher than 0.5: these include ENO2, HILPDA, and CHI3L1; BCAN was significantly downregulated in the same niche with logFC lower than -0.5. Unlike with the perinecrotic niche, the perivascular niche showed greater variability between samples, and only weak correlations on the few overlapping DEG (data not shown, see Additional file 11: Fig. S11). To get an overall idea of the DEGs in our regions of interest, we also performed DEGs analysis on the Visium data without adjusting for cell types. The top DEGs have quite a few overlaps with the result of the DEGs analysis with adjustment of cell types, although the magnitude of differential expression (logFC) is smaller after the adjustment (Additional file 12: Fig. S12).

**Fig. 4:**
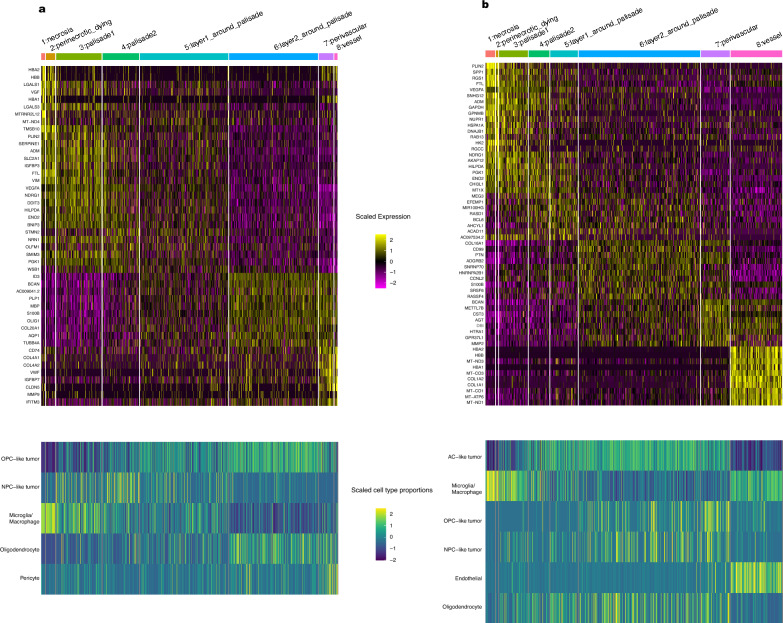
**a**, **b** Heatmaps showing the top DEGs (top) and localizations of cell types or tumor cell states (bottom) in each of the eight annotated regions, cell type adjusted, for samples 18-0282 and 19-0142, respectively. Each column represents a spot in the corresponding region. If a gene is differentially expressed in multiple regions, it’s only shown once (See Additional file 21: Table S4 for the full DEGs)

Next, we sought to characterize all the spatial regions on the slide, moving from the palisading necrosis niche to the perivascular niche. We termed this analysis “gradient analysis”. We annotated the spatial spots for each GBM sample by a gradient of regions, starting from the necrosis and radiating outward towards the vessels (Additional file 3: Fig. S3). This annotation is different from the niche annotation since it breaks down the spatial regions into smaller segments and offers a more fine-grained layer of annotation. After adjusting for cell types, we identified the top DEGs by logFC in each region (Additional file 21: Table S4). Interestingly, several genes exhibit a pattern of gradient gene expression change as we move from the necrosis towards the vessel (upper panel of Fig. 4a, b). As expected, VEGFA expression is highest near the palisades in both samples (Fig. 3). Additionally, the expression of the enzyme PGK1 inversely correlates with the distance from the palisades, reflecting its role in glycolysis. Like the DEGs analysis, we also performed the gradient analysis without adjustment of cell types. Notably, the top DEGs for each region turned out to be quite similar to the analysis result after adjusting for cell types (Additional file 13: Fig. S13).

Having observed distinct spatial localization patterns for different cell types and tumor cell states, we assessed whether these cell types and cell states preferentially localize within specific regions on our annotated slides. Notably, OPC-like tumor cells tend to be better represented in the areas closest to the vessels, i.e. perivascular region and layer2 around palisade (bottom panel of Fig. 4a, b, Additional file 14: Fig. S14). Meanwhile, microglia and macrophages are most abundant in the necrotic, perinecrotic regions, and vessel-adjacent regions, which aligns with prior expectations of macrophage behavior. Interestingly, our conclusion is consistent with prior literature [22] where they showed that GBM tumor core region (region with the highest tumor cell density) is enriched for OPC cell states. In their studies, these tumor core regions were frequently located near vessels. In our samples, the tumor-rich regions likewise coincide with regions with high OPC-like tumor representation (Additional file 14, 15: Figs. S14, S15).

We subsequently used GO Enrichment Analysis [8] and IPA [12] to identify pathways of interest throughout the tumor as we moved from palisading necrosis towards vessels (GO results in Additional file 22: Table S5). In the case where we adjusted for cell types (Additional file 16: Fig. S16), the most marked differences came in the form of energy consumption: Glycolysis I and Gluconeogenesis were highest in the necrotic and perinecrotic regions, while Oxidative Phosphorylation was highest in perivascular regions. Markers of cell stress followed a similar pattern: Ferroptosis, the Unfolded Protein Response, and Chaperone-mediated Autophagy were all elevated in the necrotic and perinecrotic regions. In a parallel manner, we found that the necrotic and perinecrotic regions were generally more immunosuppressive even relative to the globally immunosuppressive glioblastoma microenvironment. We repeated the analyses without adjusting for cell types, to better assess the global gene expression changes. The pathways identified by IPA were largely similar regardless of whether we adjusted for cell types or not. (Additional file 17: Fig. S17).

## Discussion

Despite decades of research to understand the molecular underpinnings of GBM, the median survival time is short and the standard of care for the disease remains limited. Single-cell technologies have revolutionized the characterization of GBM tumor heterogeneity and plasticity [17, 25]. However, few studies have evaluated the spatial organization of cell types and tumor cell states in GBM with matched single-cell and spatial transcriptomics data. Here, we integrated paired snRNA-seq and spatial transcriptomics data from three GBM patients to uncover key signaling pathways in the GBM microenvironment. Our analyses offer insights into the landscape of the preferential localizations of different cell types and tumor cell states, as well as an illustration of gene expression and pathways in the gradient of regions within GBM.

Our copy number analysis shows that each GBM patient has distinct patterns of copy number alternations, although all samples demonstrated the canonical GBM gain of chromosome 7 and loss of chromosome 10, by both inferCNV and chromosomal microarray results (Fig. 1c, Additional file 4: Fig. S4). Through deconvolution of cell states within the GBM tissues, we observe that in most cases, the four developmental states of GBM cells (NPC-like, OPC-like, AC-like and MES-like tumors) localize to distinct regions of the tumor, appearing to form their own niches (Fig. 2a-c). For example, in sample 19-0341, AC-like tumor and OPC-like tumor significantly segregate. In samples 18-0282 and 19-0341, which both show a relatively high abundance of OPC-like tumor, OPC-like and NPC-like tumor also significantly segregate. Hence, at least in cases with significant populations of tumor cells from differing cell states, the different cell states of tumor tend to segregate. While histologic intratumoral heterogeneity is a well-recognized feature of GBM, this supports the additional presence of cell-state heterogeneity within a tumor, with different tumor cell states segregating to establish their unique niches. Our results agree with previous findings [17], which posit that different tumors exhibit widely variable distribution of cell states, but goes further in demonstrating that the cell states are not evenly distributed throughout a tumor but appear to segregate into distinct niches. Further studies in larger cohorts are needed to identify the key signaling driving maintenance of each cell state, and research into therapeutic approaches should consider potential differential impacts on the different cell states.

Our gradient analysis provides a characterization of the spatial regions in GBM microenvironment. We revealed the cell type localizations and pathways-of-interest in each of the curated regions from the palisading necrosis niche to the perivascular niche. The preferential localization of OPC-like tumors in the perivascular niche and in areas closer to the tumor vasculature was especially notable. Non-tumor OPCs are dependent on access to blood supply to avoid hypoxia [28]; in non-tumor OPCs, hypoxia can induce premature maturation [1]. It is likely that the localization of OPC-like tumor cells near blood vessels may reflect the cellular requirements for elevated oxygen levels for the maintenance of an OPC-like state. From IPA, we found that the perinecrotic regions were enriched for immunosuppressive signaling pathways such as IL-10 and Granzyme A Signaling. Even within tumors which are globally immunosuppressed, this demonstrated variability in degree of immunosuppression within the tumor microenvironment. Notably, we observed that macrophages are enriched in the necrosis and perinecrotic regions (Fig. 4a, b). Thus, it is highly likely that these macrophages are especially immunosuppressive within the globally immunosuppressed tumor. Despite the small sample size, our study reveals important findings in the GBM TME. Rather than using public datasets for deconvolution of our spatial data, our use of matched snRNA-seq with spatial transcriptomics data led to more robust integration of the results, since the two data modalities are derived from the same tissue.

In conclusion, we have defined the spatial localization of different cell types and tumor cell states in the GBM microenvironment, as well as uncovered significant gene targets and pathways near the perivascular and palisading necrosis niches. While further studies will be required to validate some of our findings, we have made significant progress in defining the spatial context of cell types and cell states within heterogeneous GBM tumors. Importantly, we identified patterns of segregation between different cell states of tumor and revealed marked immunosuppressive features within the perinecrotic niche.

### Supplementary Information

**Additional file 1: Fig. S1**. UMAP of the snRNA-seq data by sample of origin and by cell states per sample, after batch correction with Seurat.

**Additional file 2: Fig. S2**. Clustree analysis to determine the resolution to use when clustering snRNA-seq data. A resolution of 0.5 was chosen because as the resolution gets higher than 0.5, there are too many clusters with multiple incoming edges and thus we’ve over-clustered.

**Additional file 3: Fig. S3**. Annotation of the gradient of regions for samples 18-0282 and 19-0142, respectively.

**Additional file 4: Fig. S4**. Chromosomal microarray results for samples 18-0282, 19-0142 and 19-0341 respectively.

**Additional file 5: Fig. S5**. The counts of each cell state/cell type in the snRNA-seq data from the same block as the spatial transcriptomics data for samples 18-0282, 19-0142 and 19-0341 respectively.

**Additional file 6: Fig. S6**. Cell type correlation plots for samples 18-0282, 19-0142 and 19-0341, respectively.

**Additional file 7: Fig. S7**. Annotation of the niches of interest for samples 18-0282 and 19-0142, respectively.

**Additional file 8: Fig. S8**. Higher resolution H&E images for 18-0282.

**Additional file 9: Fig. S9**. Higher resolution H&E image for 19-0142.

**Additional file 10: Fig. S10**. Higher resolution H&E image for 19-0341.

**Additional file 11: Fig. S11**. Plot for samples 18-0282 and 19-0142, logFC of gene expression in perivascular niche compared to the generic tumor region, cell type adjusted. Whether the logFC of the gene is significant is shown in the legend. Adjusted *p* values < 0.05 were labeled as significant.

**Additional file 12: Fig. S12**. Without adjustment of cell types, plots for samples 18-0282 and 19-0142, logFC of gene expression in a) palisading necrosis niche compared to the generic tumor region, and b) perivascular niche compared to the generic tumor region. Whether the logFC of the gene is significant is shown in the legend. Adjusted *p* values < 0.05 were labeled as significant.

**Additional file 13: Fig. S13**. Without adjustment of cell types, heatmaps showing the top DEGs in each of the eight annotated regions, for samples 18-0282 and 19-0142 respectively. Each column represents a spot in the corresponding region.

**Additional file 14: Fig. S14**. Boxplots showing the enrichment of OPC-like tumor in each region, for samples 18-0282 and 19-0142 respectively.

**Additional file 15: Fig. S15**. Gradient analysis combining different tumor cell states, showing the enrichment of cell types in each gradient of regions, for samples 18-0282 and 19-0142 respectively.

**Additional file 16: Fig. S16**. Significant pathways in each annotated region, adjusted for cell types, revealed by Ingenuity Pathway Analysis (partial results), FDR < 0.05. Warmer/cooler colors are up/downregulated pathways.

**Additional file 17: Fig. S17**. Significant pathways in each annotated region, not adjusted for cell types, revealed by Ingenuity Pathway Analysis (partial results), FDR < 0.05. Warmer/cooler colors are up/downregulated pathways.

**Additional file 18: Table S1**. Clinical characteristics and sample information (number of spots, genes, reads per sample).

**Additional file 19: Table S2**. Summary of the chromosomal microarray results for samples 18-0282, 19-0142 and 19-0341 respectively.

**Additional file 20: Table S3**. Percentage of cell states per cluster, and the calculated mean lineage scores for each cell state per cluster (related to “Cell type and cell state annotation for snRNA-seq” in Materials and methods).

**Additional file 21: Table S4**. Differentially expressed genes in gradient analysis, cell type adjusted (related to Fig. 4).

**Additional file 22: Table S5**. GO enrichment analysis results for perivascular and perinecrotic niches for samples 18-0282 and 19-0142, cell type adjusted.

## Data Availability

The datasets generated and analyzed during the current study are in the process of being deposited in National Center for Biotechnology Information database of Genotypes and Phenoptypes (NCBI dbGAP). High resolution H&E images from each case can be found at 10.7924/r4nk3k26s. Notes: Data were analyzed through the use of IPA (QIAGEN Inc., https://www.qiagenbioinformatics.com/products/ingenuitypathway-analysis). Software versions: 10x Genomics Cell Ranger 7.0.0; 10x Genomics Space Ranger 1.2.1; 10x Genomics Loupe Browser 4.1.0; Qiagen Ingenuity Pathway Analysis 01-20-04.
