# Supplementary figures and images for: Spatial transcriptomics reveals segregation of tumor cell states in glioblastoma and marked immunosuppression within the perinecrotic niche
Source: Acta Neuropathol Commun. 2024 Apr 22;12:64. doi: 10.1186/s40478-024-01769-0 (PMC11036705; doi:10.1186/s40478-024-01769-0)

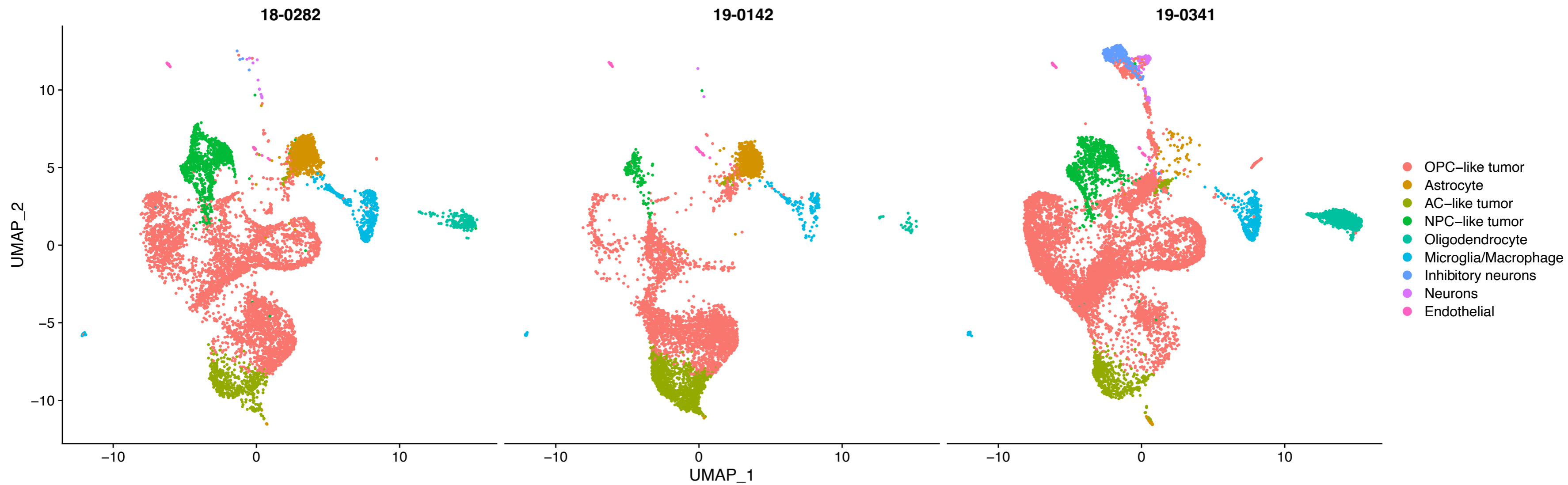

Supplement: Supplementary file 1 — Additional file 1: Fig. S1. UMAP of the snRNA-seq data by sample of origin and by cell states per sample, after batch correction with Seurat. [file 40478_2024_1769_MOESM1_ESM.pdf]

clustree analysis

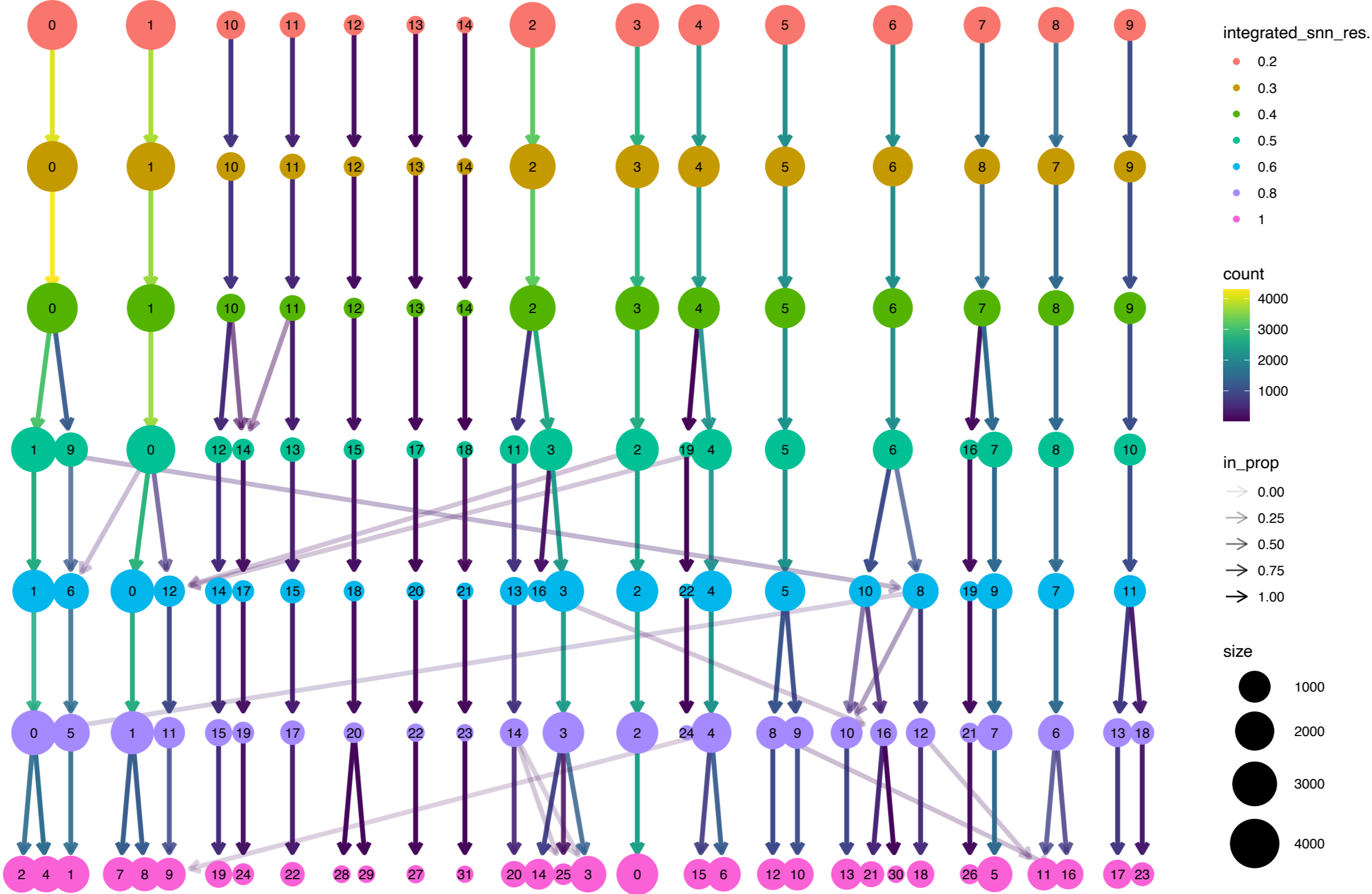

Supplement: Supplementary file 2 — Additional file 2: Fig. S2. Clustree analysis to determine the resolution to use when clustering snRNA-seq data. A resolution of 0.5 was chosen because as the resolution gets higher than 0.5, there are too many clusters with multiple incoming edges and thus we’ve over-clustered. [file 40478_2024_1769_MOESM2_ESM.pdf]

a18-0282

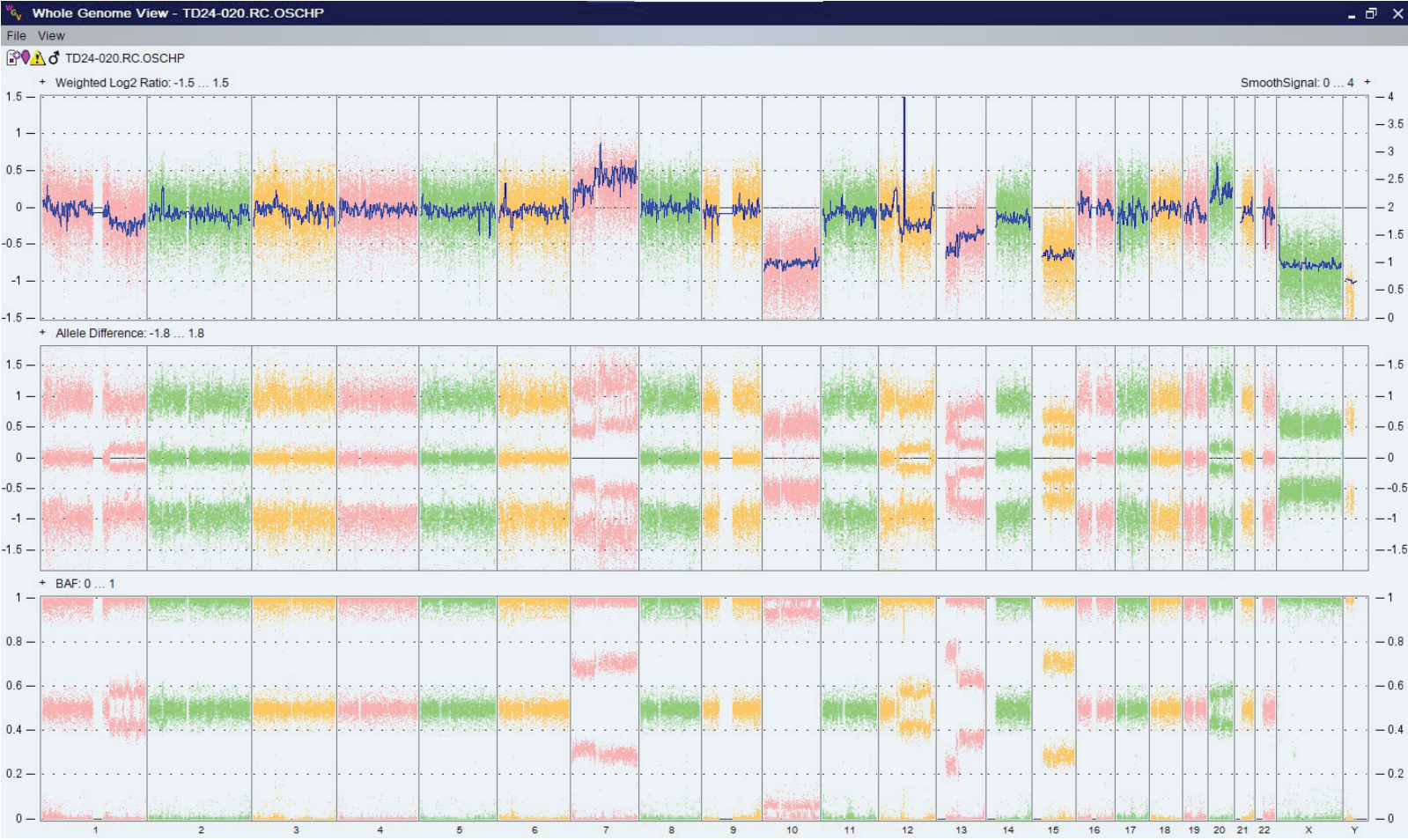

b19-0142

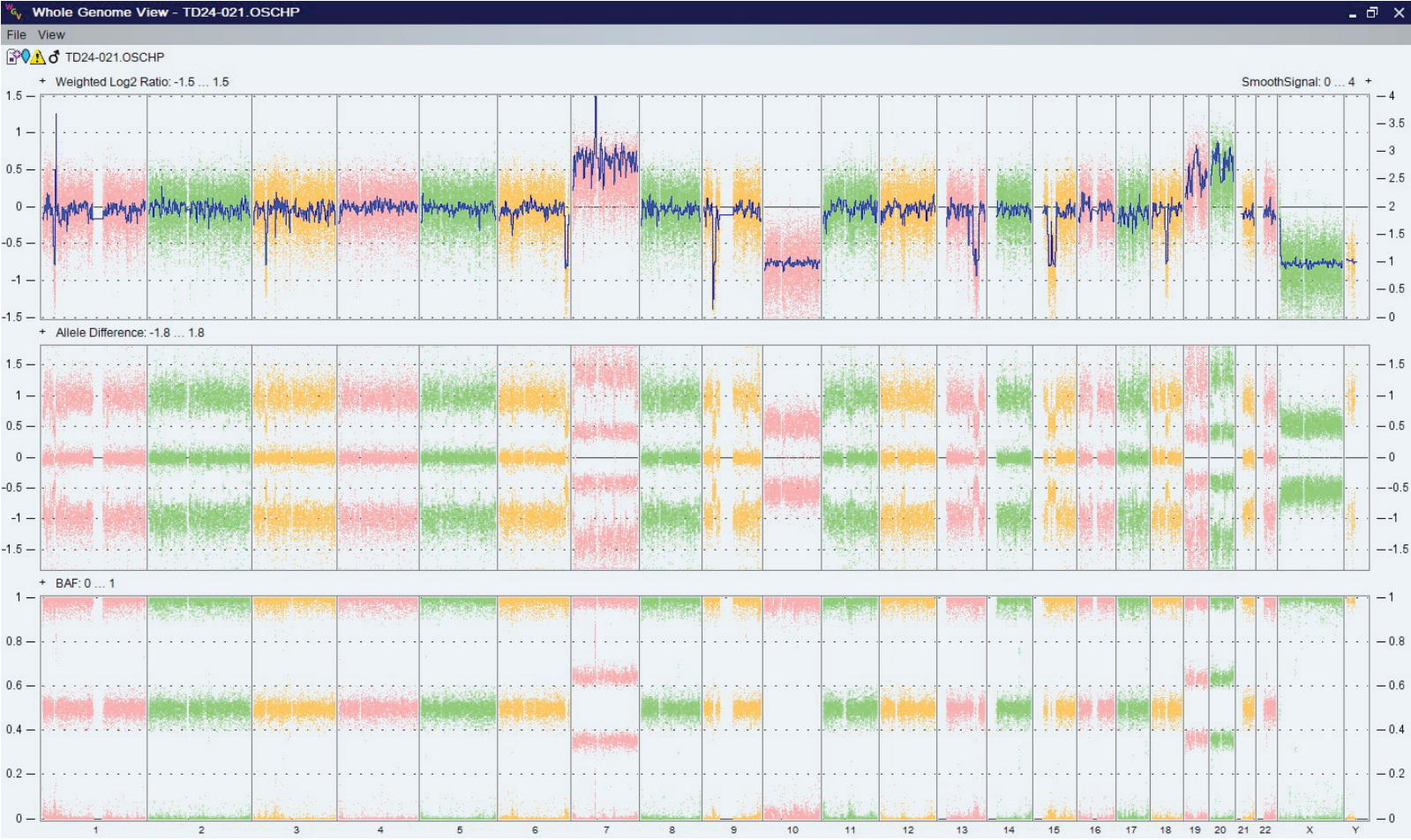

c19-0341

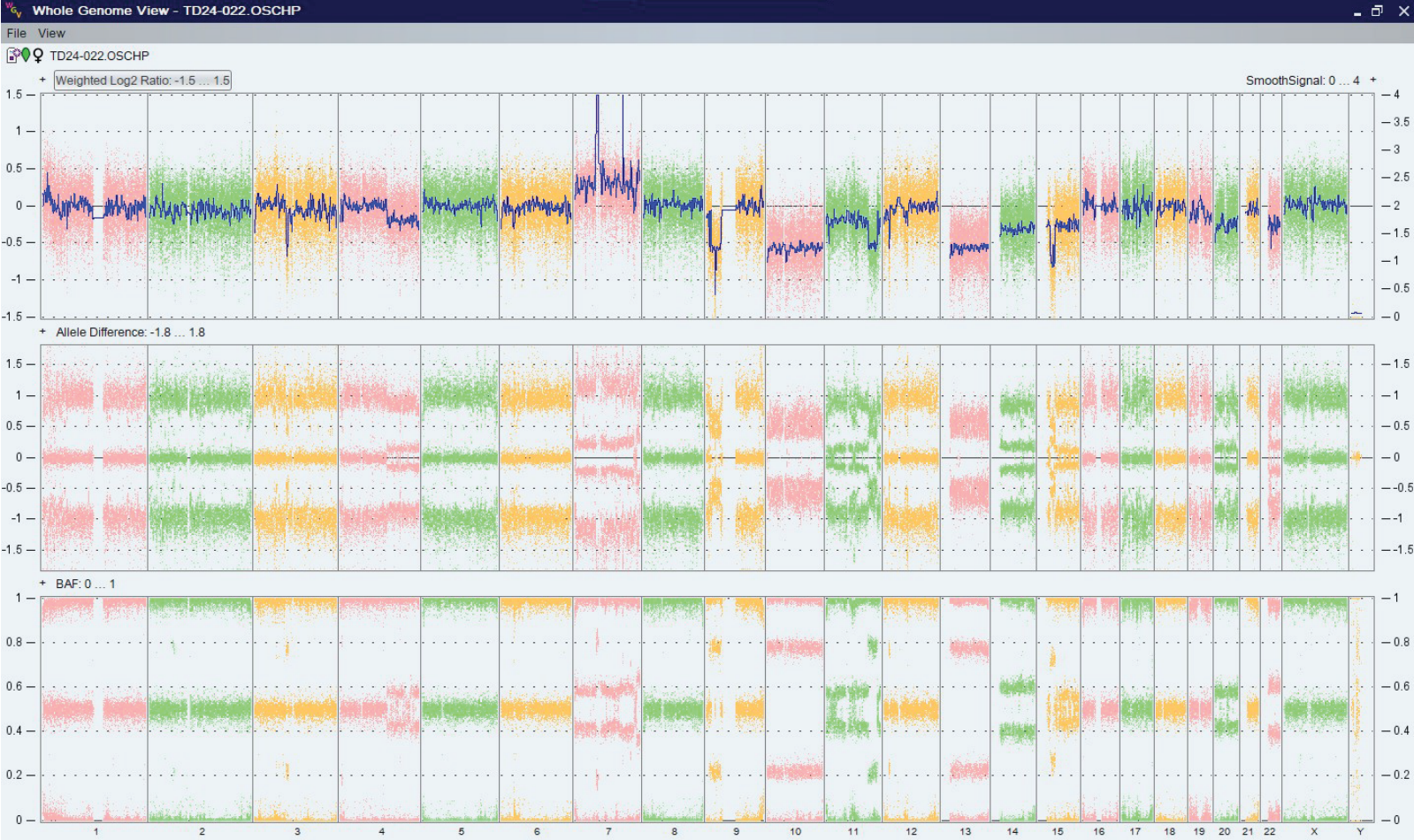

Supplement: Supplementary file 4 — Additional file 4: Fig. S4. Chromosomal microarray results for samples 18-0282, 19-0142 and 19-0341 respectively. [file 40478_2024_1769_MOESM4_ESM.pdf]

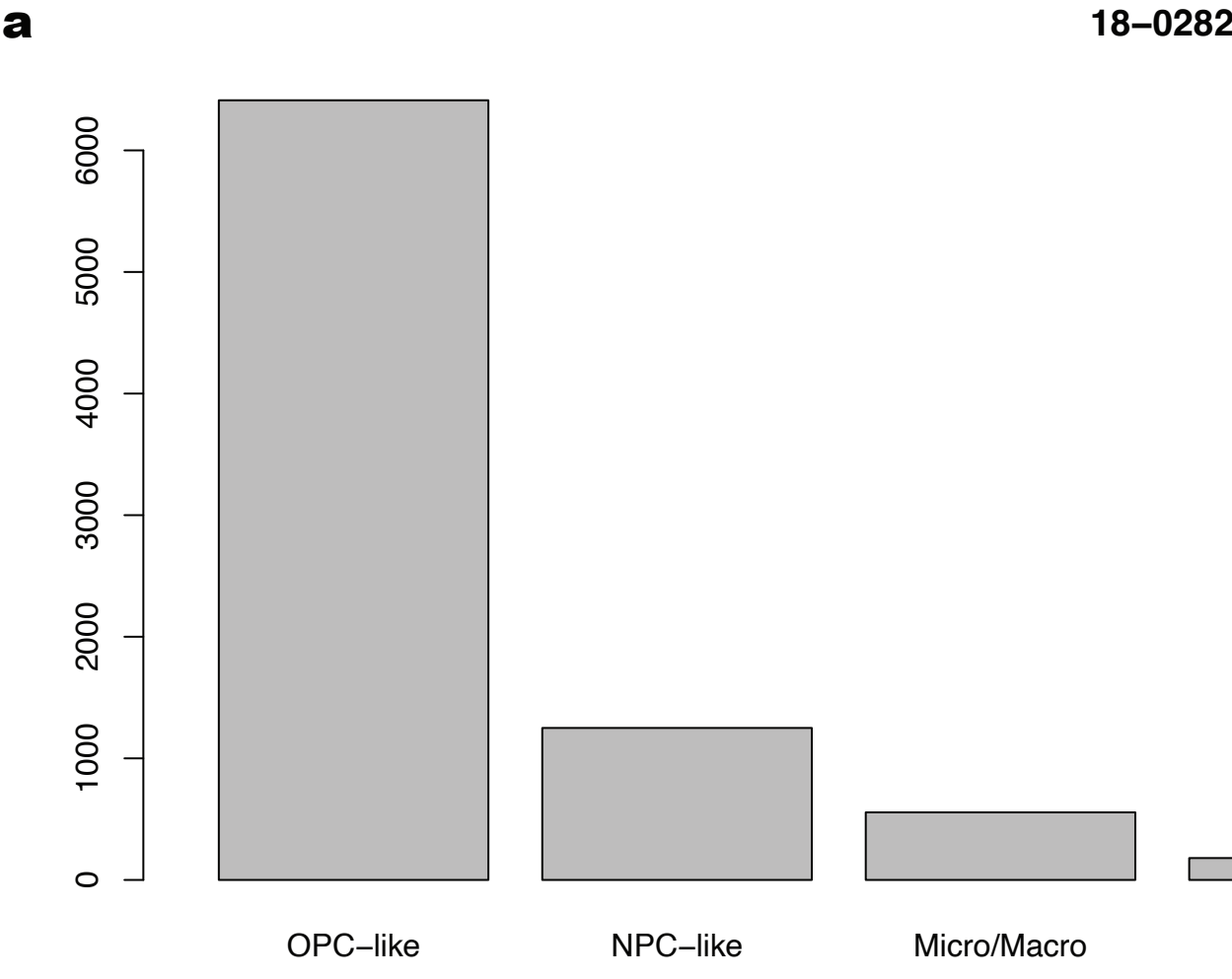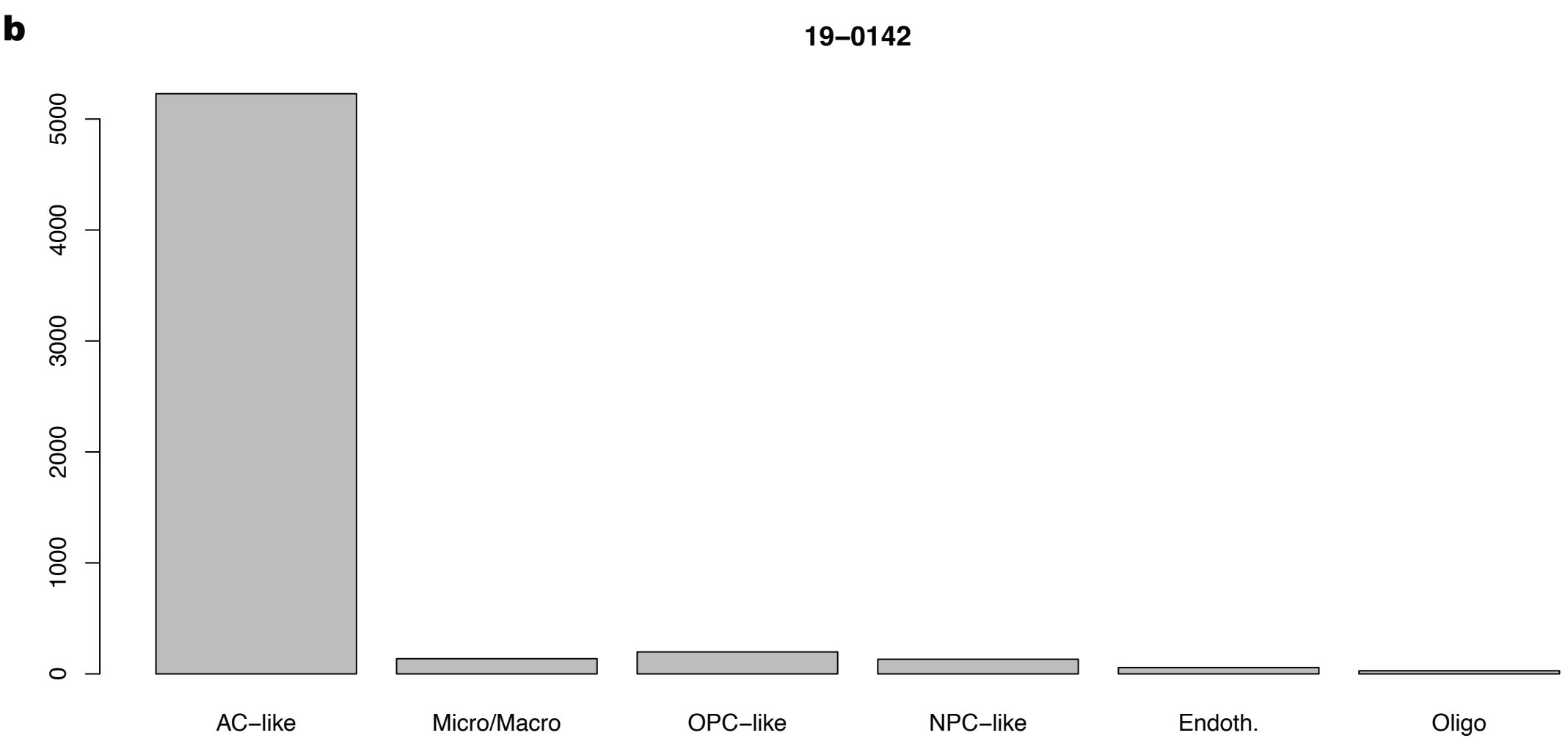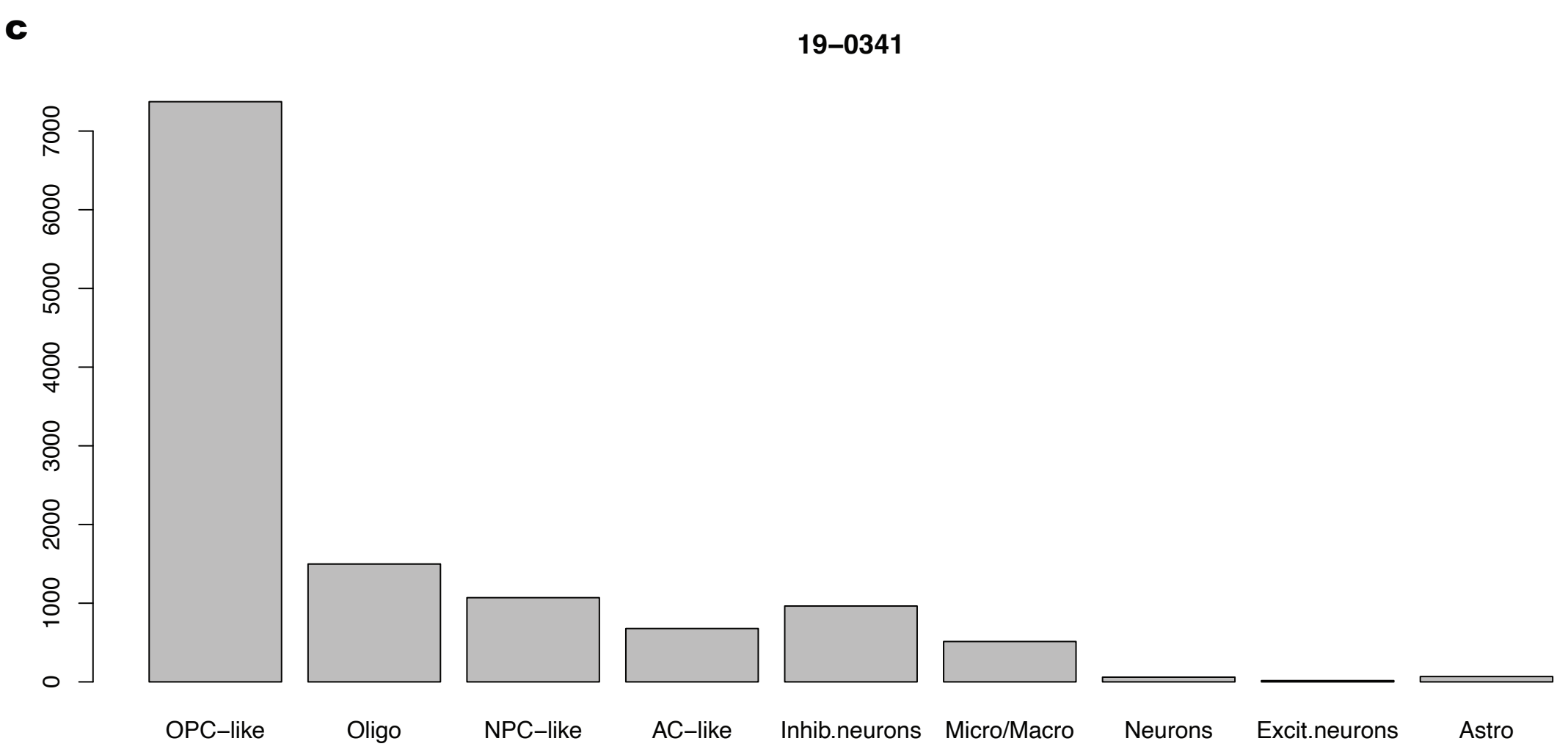

Supplement: Supplementary file 5 — Additional file 5: Fig. S5. The counts of each cell state/cell type in the snRNA-seq data from the same block as the spatial transcriptomics data for samples 18-0282, 19-0142 and 19-0341 respectively. [file 40478_2024_1769_MOESM5_ESM.pdf]

18-0282:

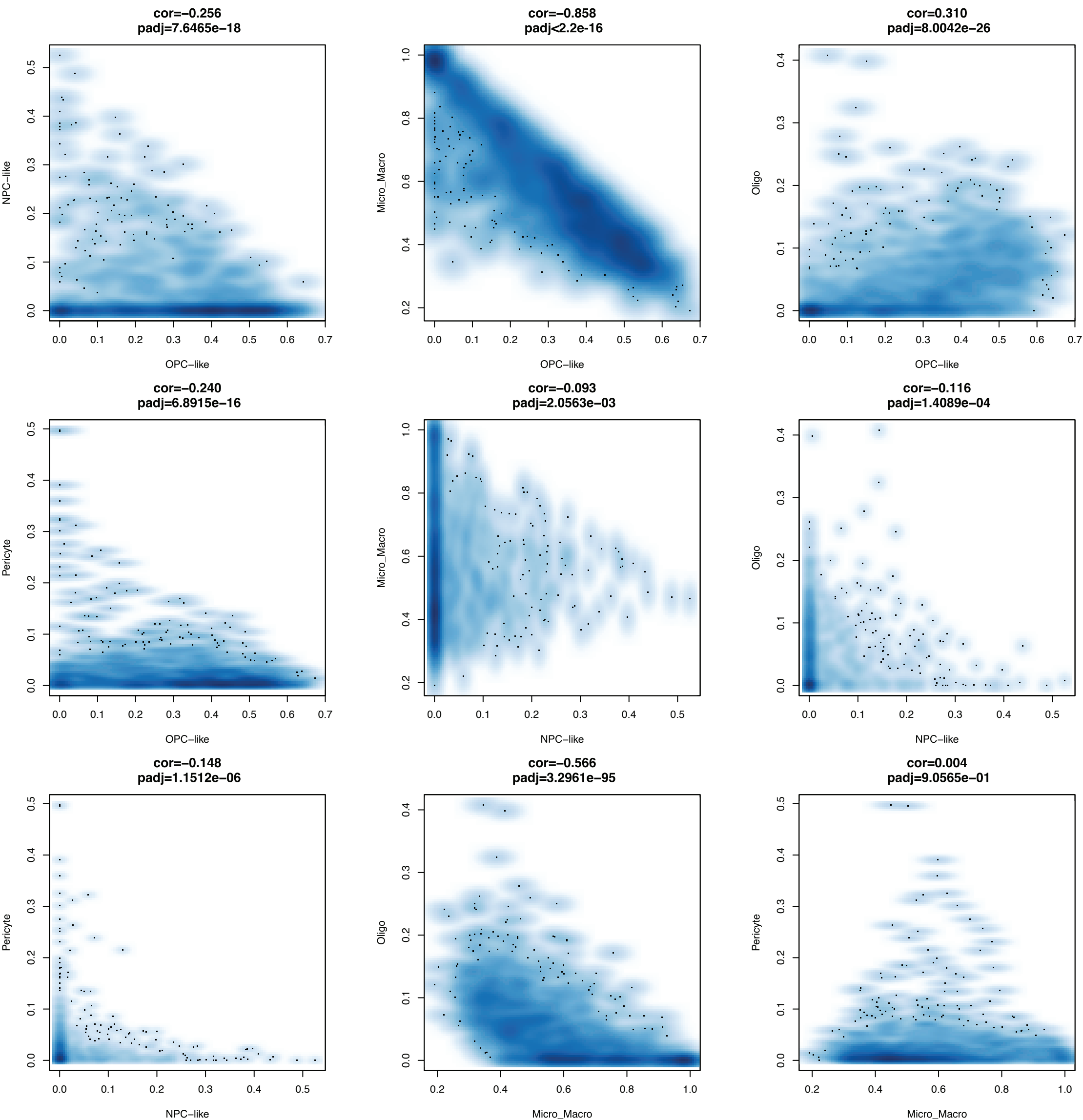

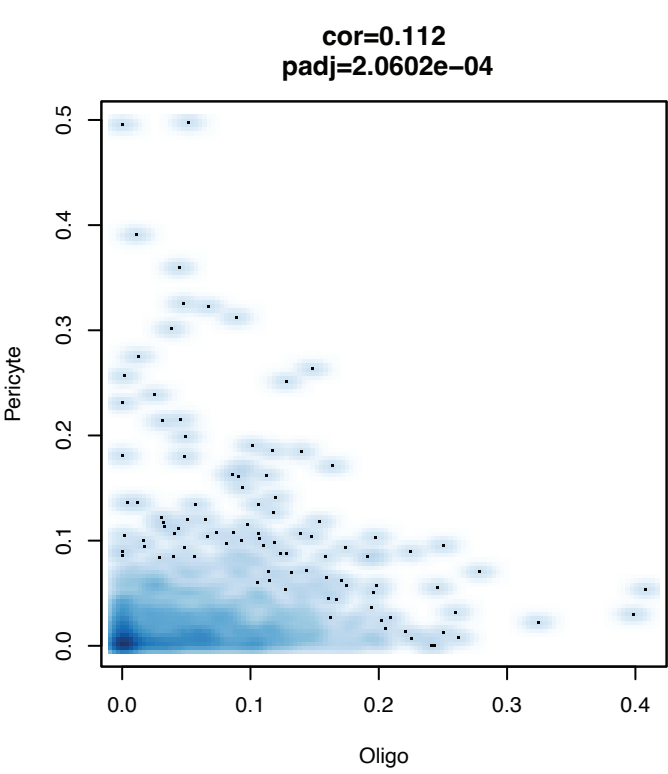

19-0142:

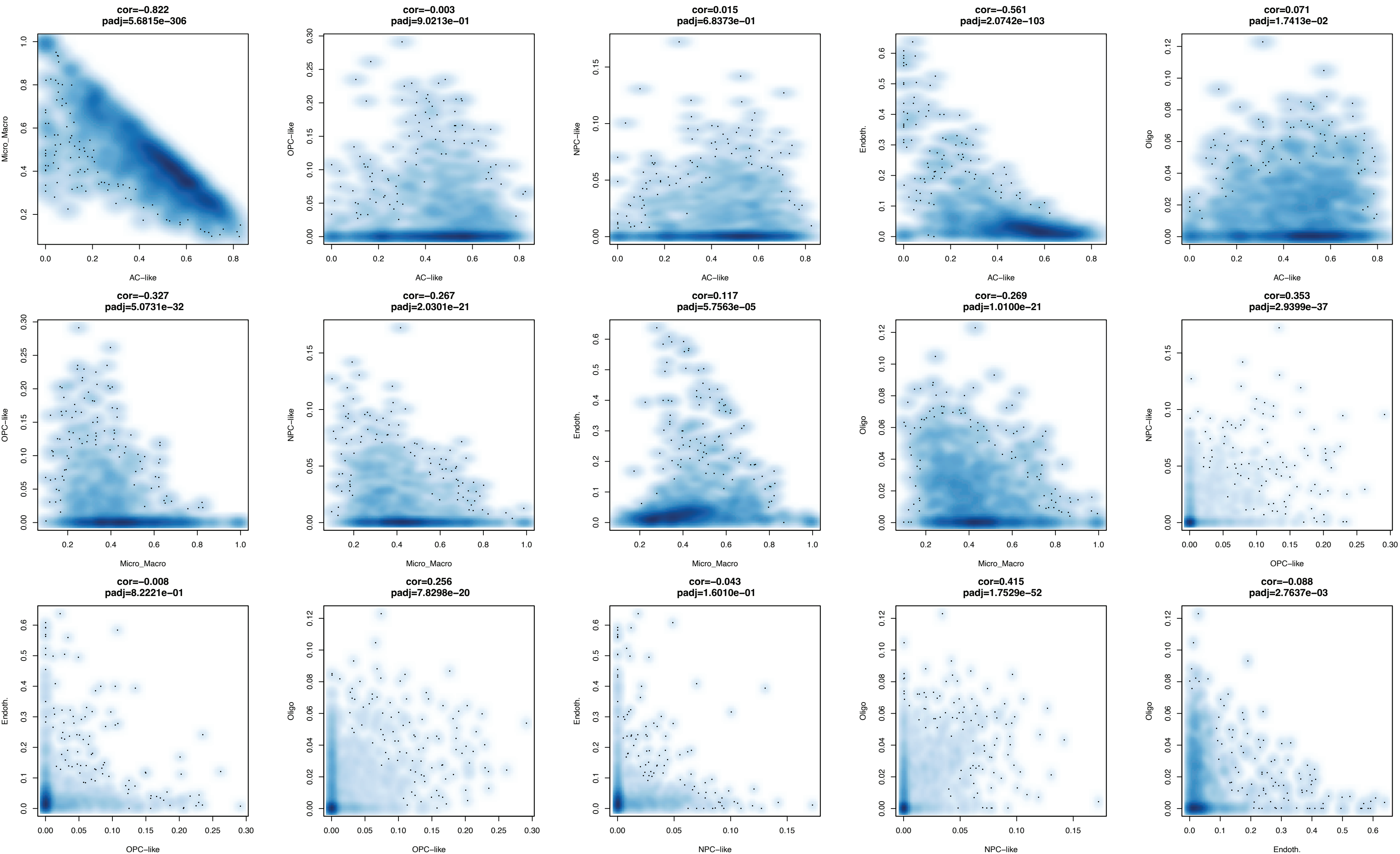

19-0341:

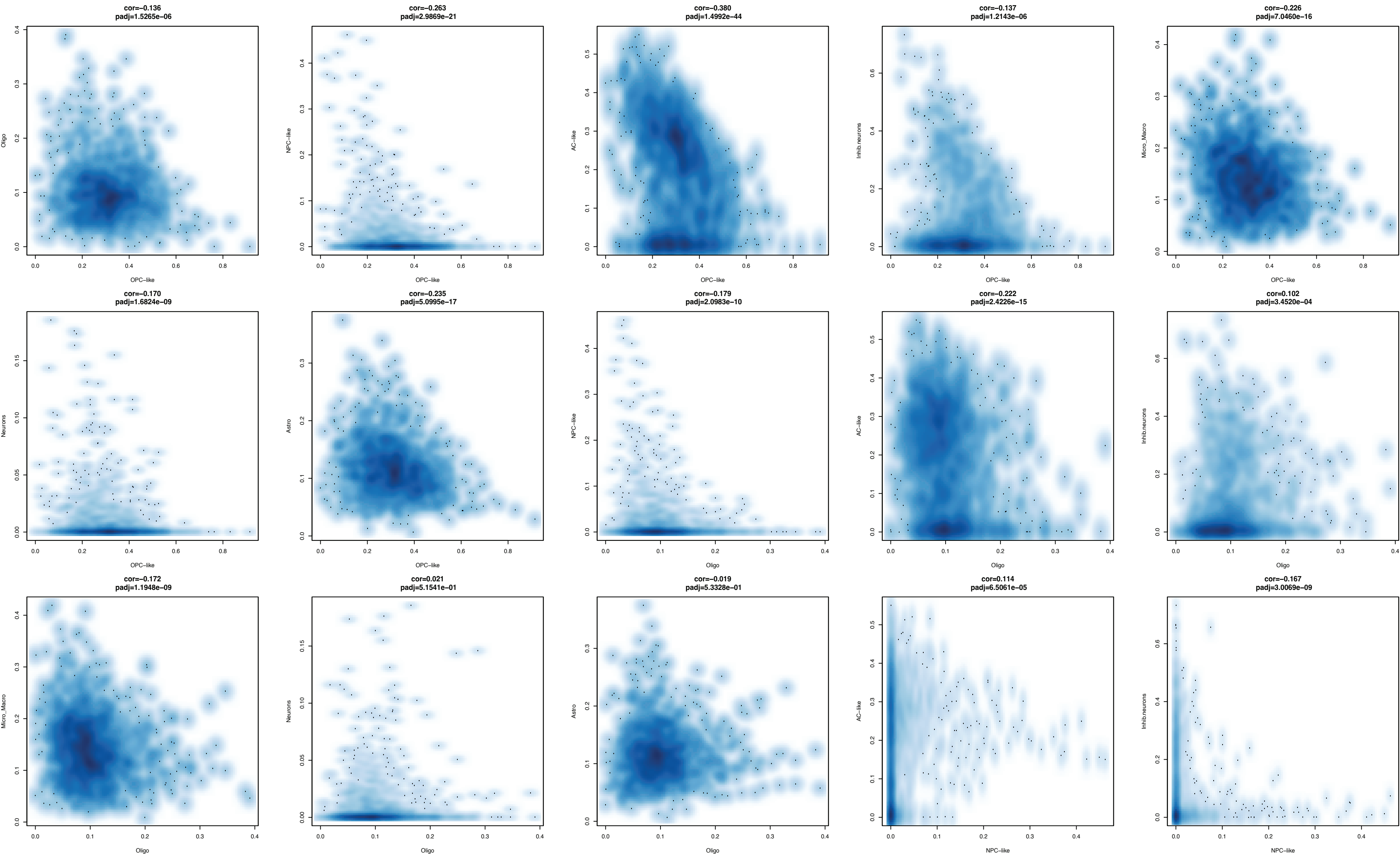

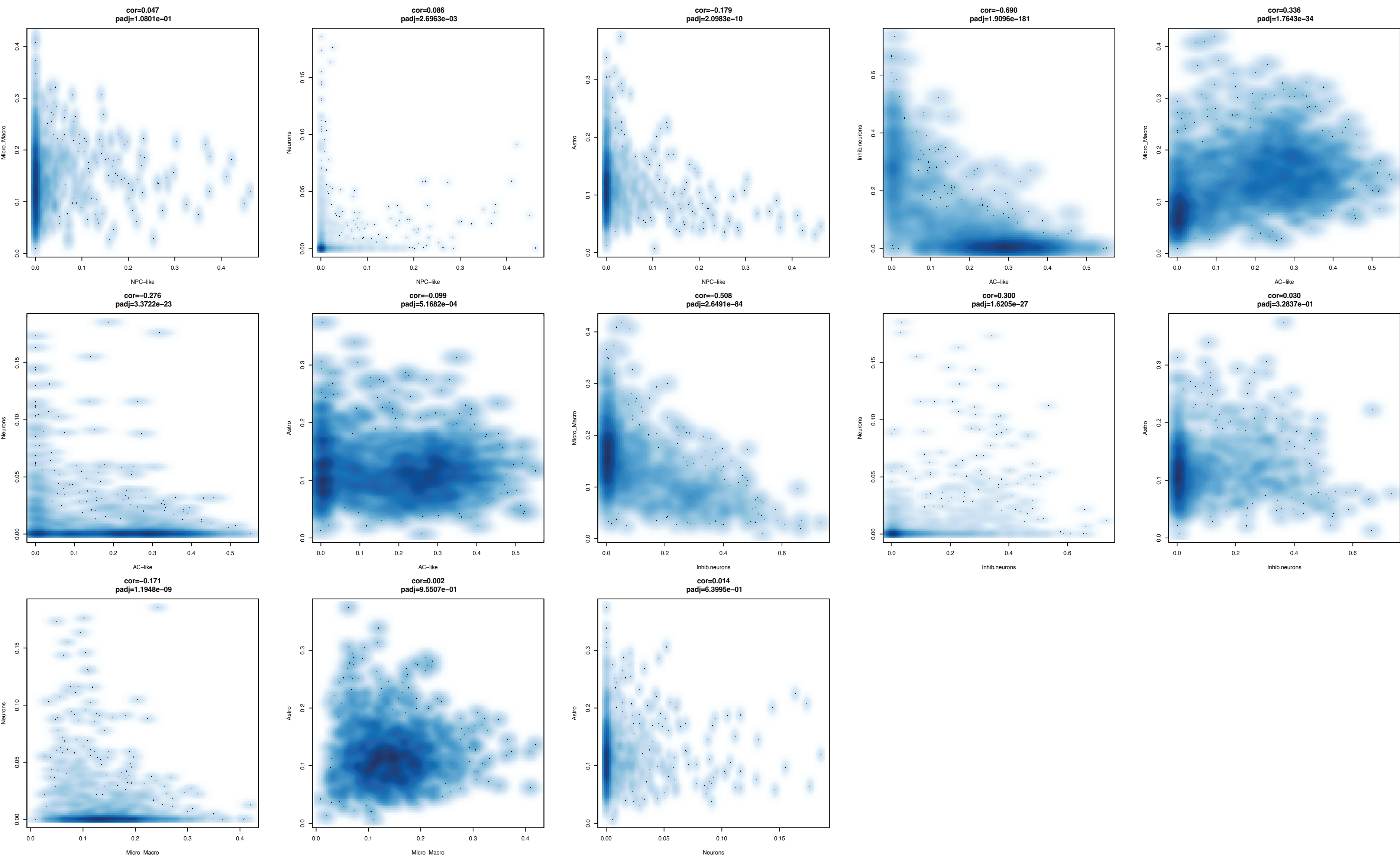

Supplement: Supplementary file 6 — Additional file 6: Fig. S6. Cell type correlation plots for samples 18-0282, 19-0142 and 19-0341, respectively. [file 40478_2024_1769_MOESM6_ESM.pdf]

**a**

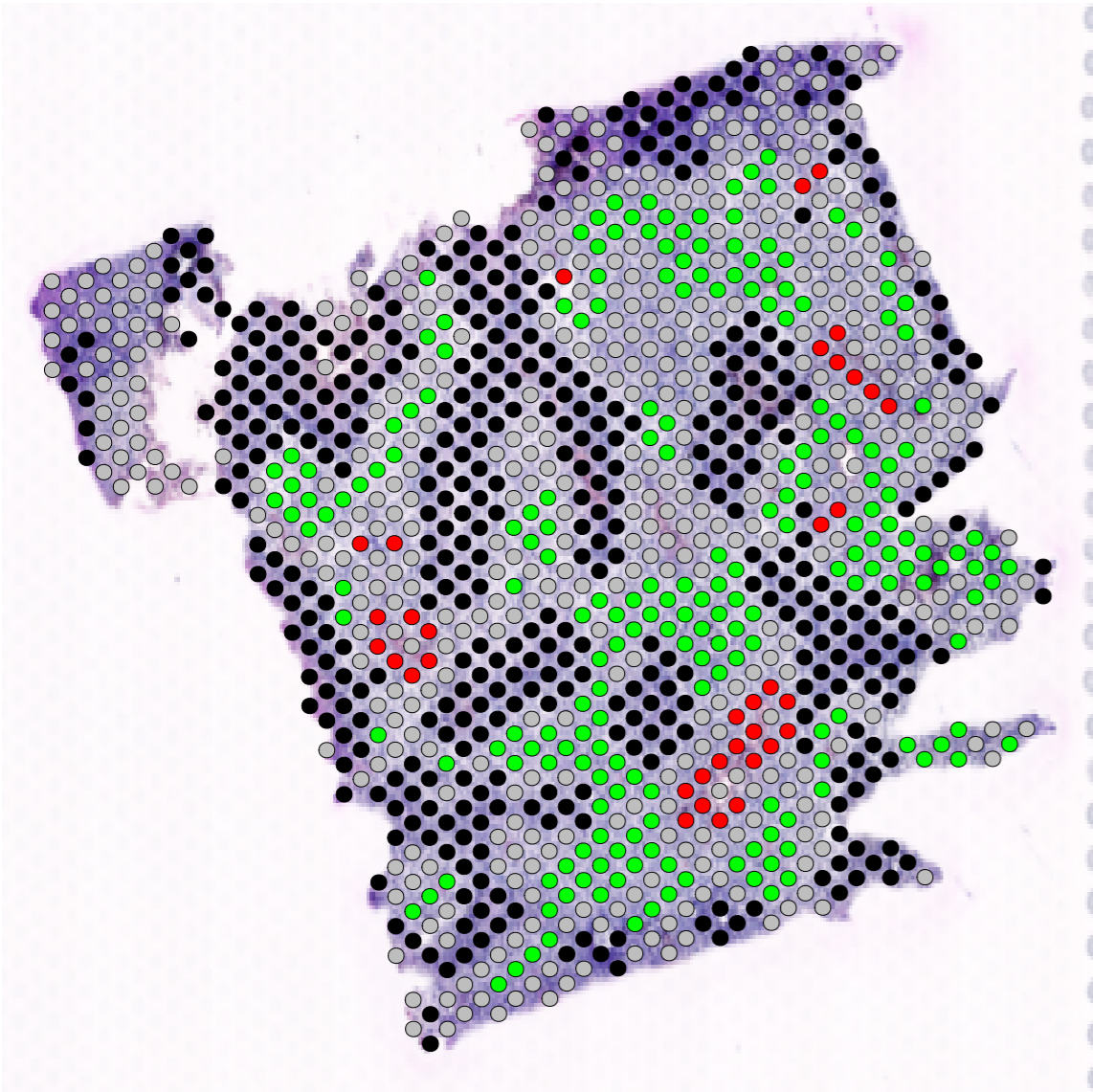

**b**

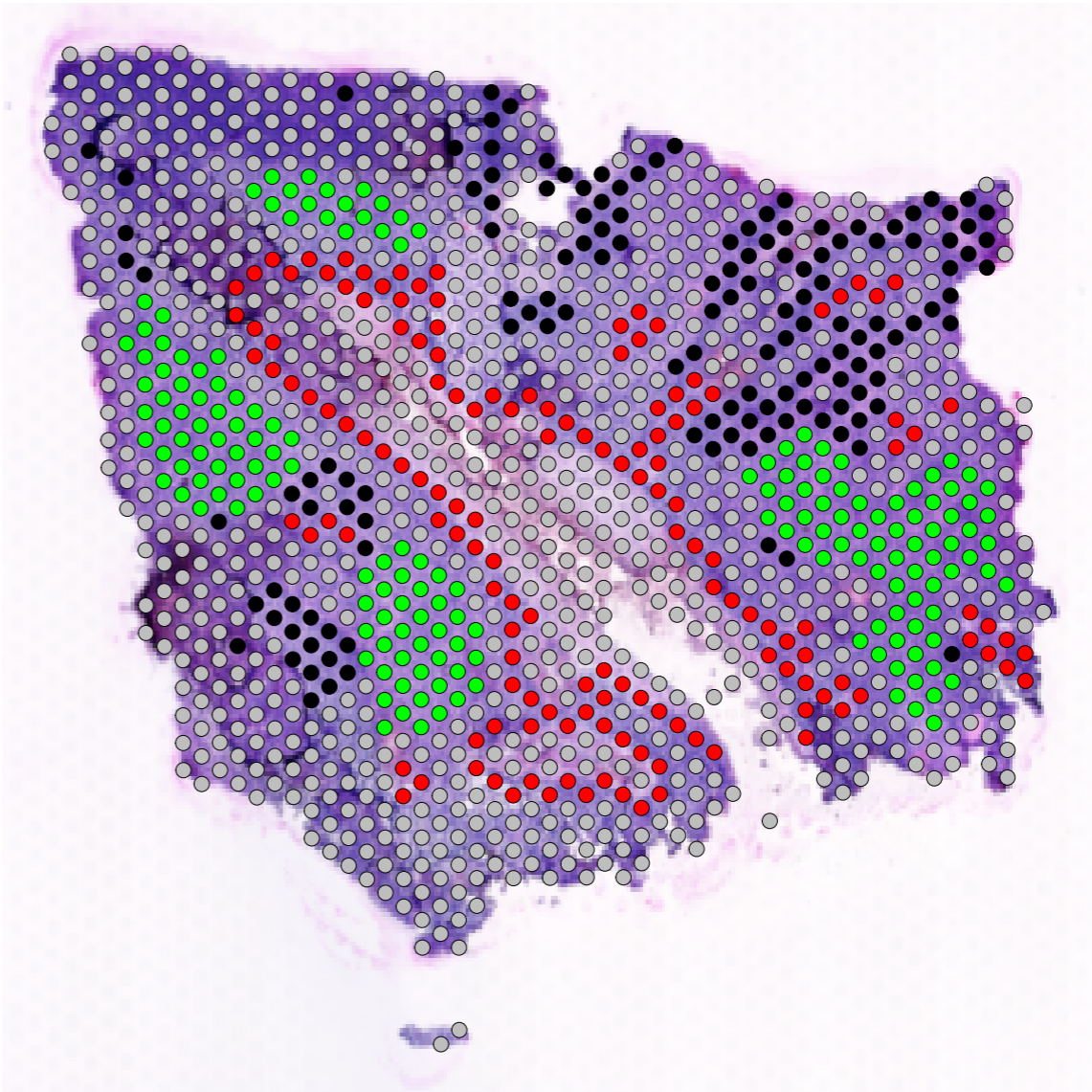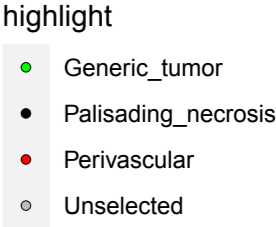

Supplement: Supplementary file 7 — Additional file 7: Fig. S7. Annotation of the niches of interest for samples 18-0282 and 19-0142, respectively. [file 40478_2024_1769_MOESM7_ESM.pdf]

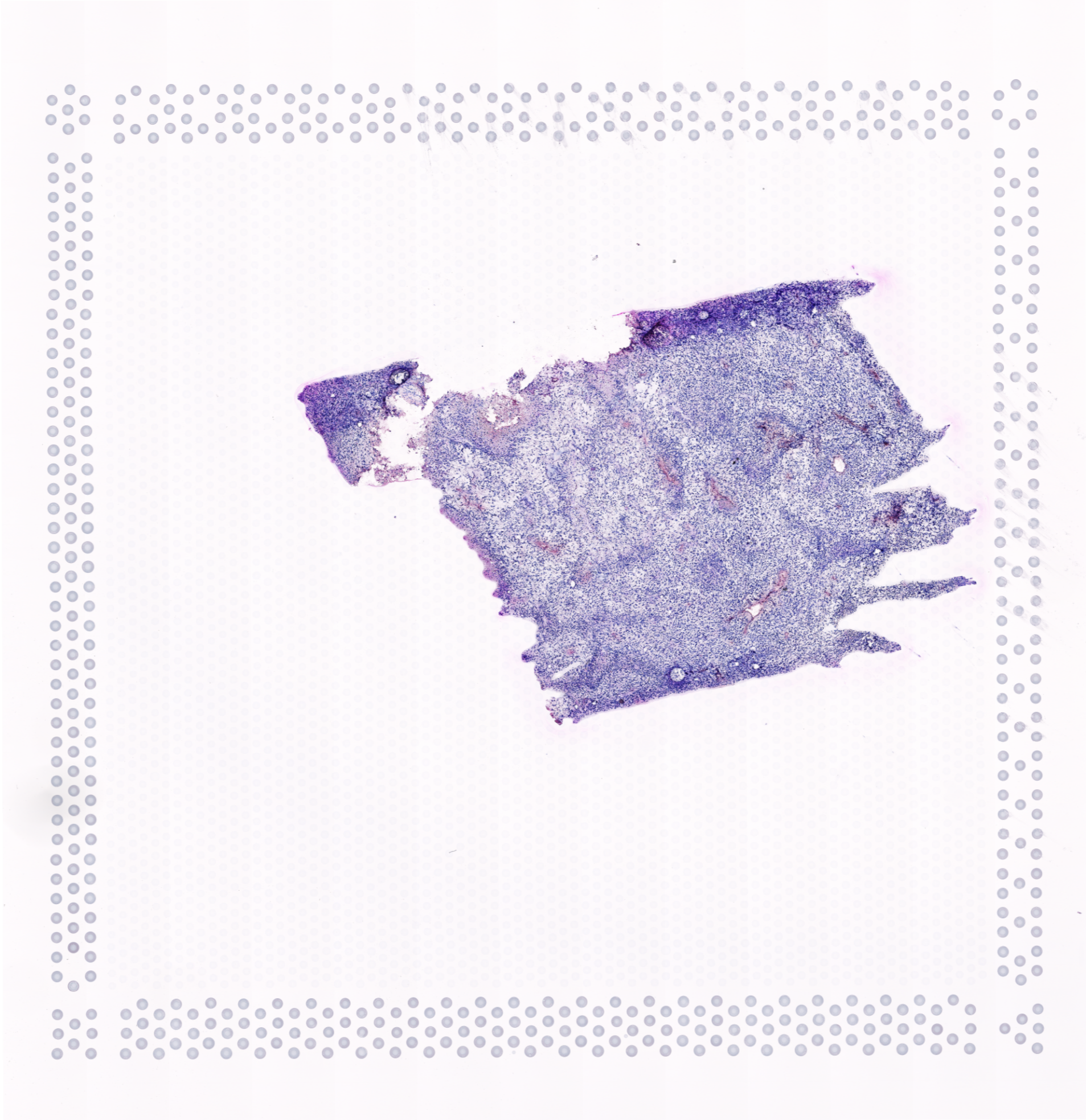

Supplement: Supplementary file 8 — Additional file 8: Fig. S8. Higher resolution H&E images for 18-0282. [file 40478_2024_1769_MOESM8_ESM.pdf]

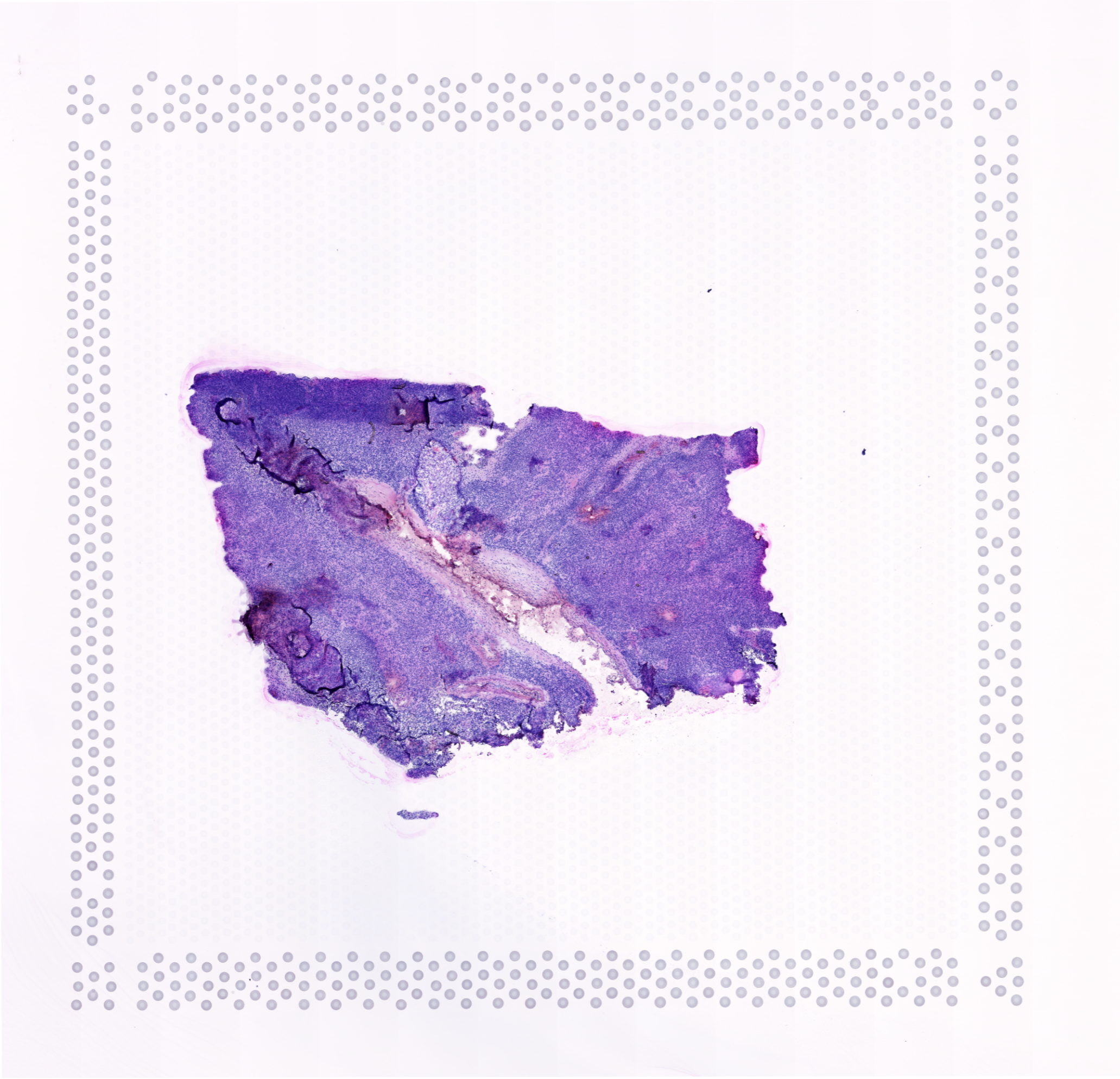

Supplement: Supplementary file 9 — Additional file 9: Fig. S9. Higher resolution H&E image for 19-0142. [file 40478_2024_1769_MOESM9_ESM.pdf]

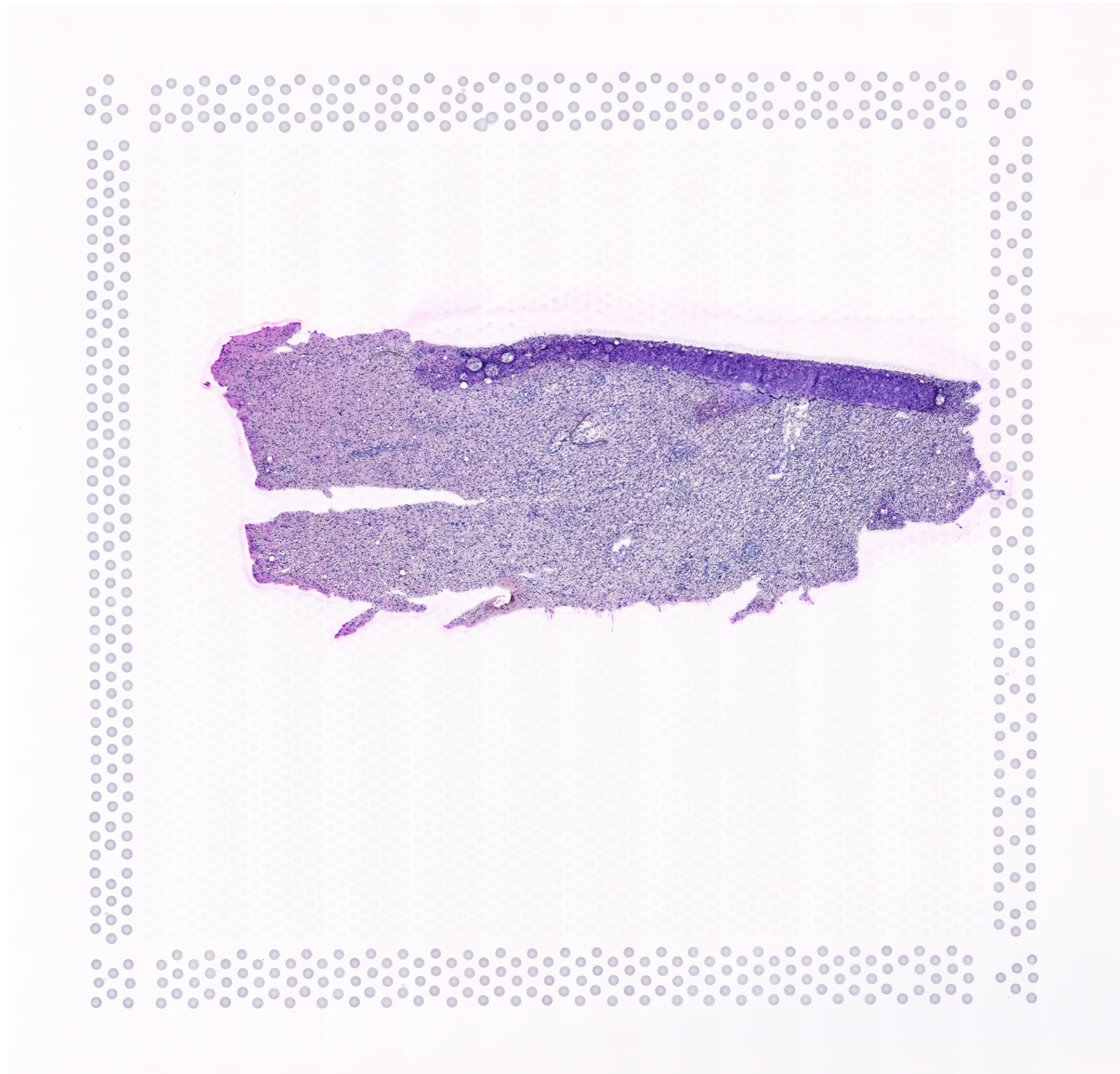

Supplement: Supplementary file 10 — Additional file 10: Fig. S10. Higher resolution H&E image for 19-0341. [file 40478_2024_1769_MOESM10_ESM.pdf]

**a**

Correlation = 0.5260

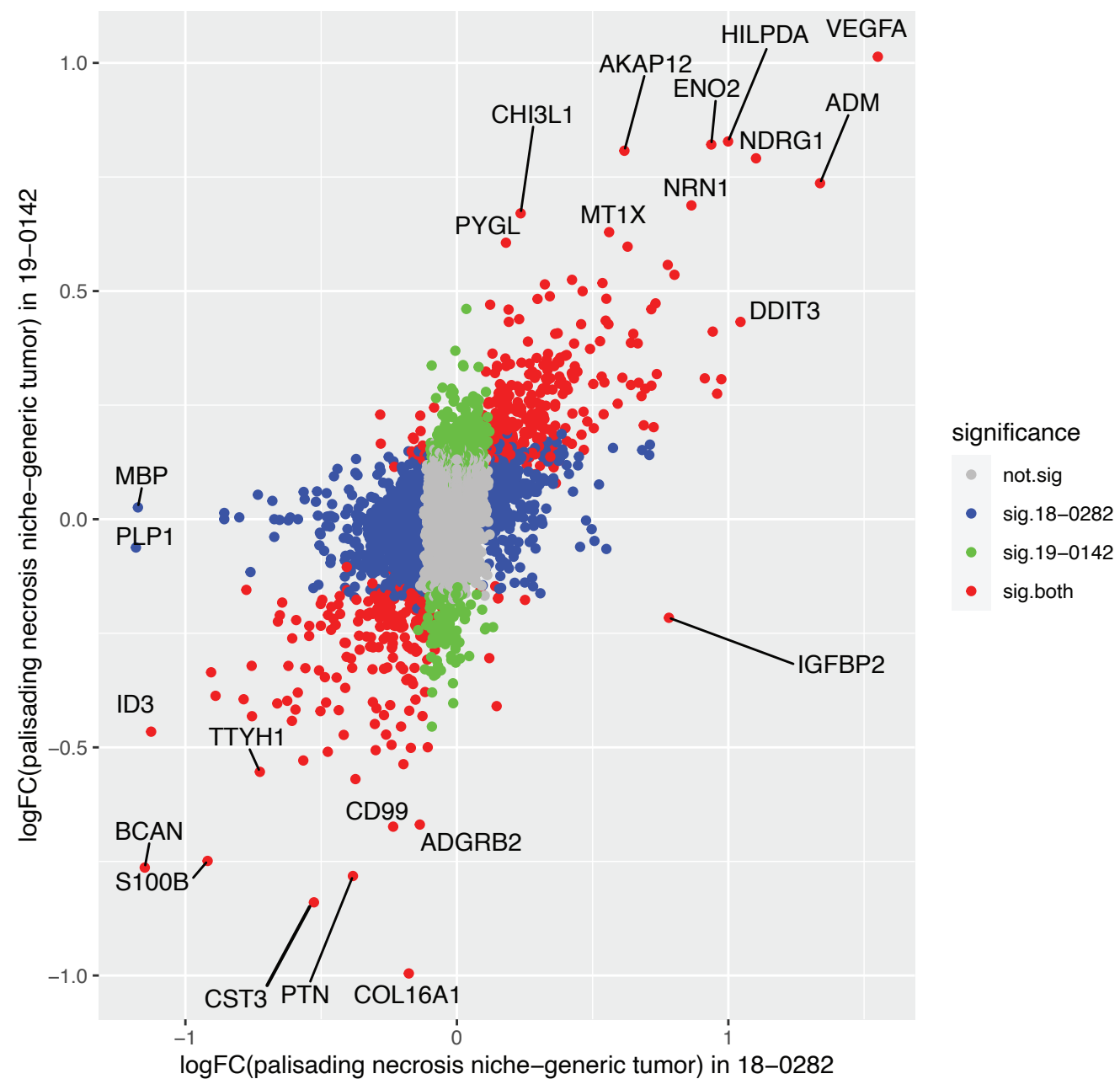**b**

Correlation = 0.1578

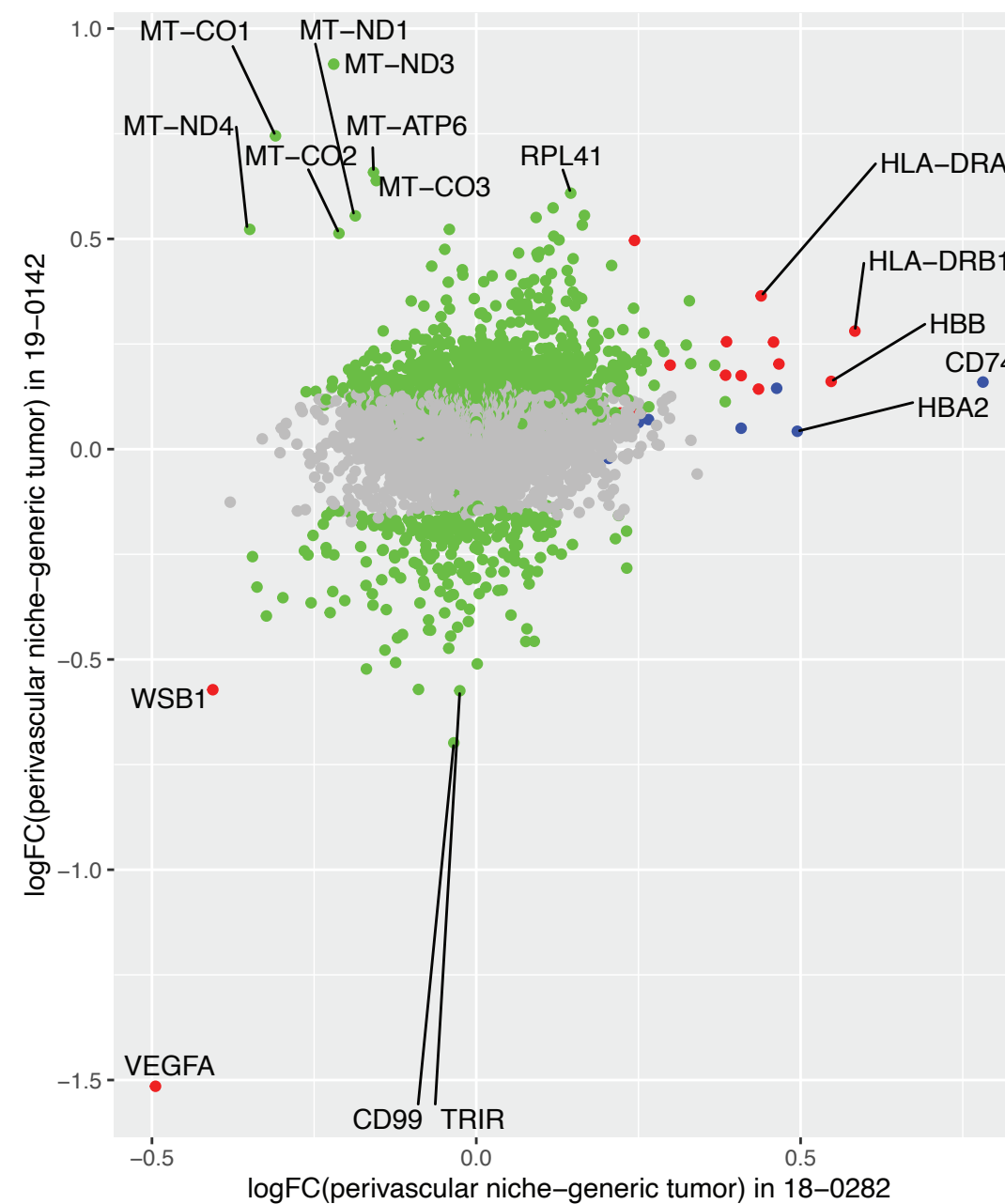

Supplement: Supplementary file 12 — Additional file 12: Fig. S12. Without adjustment of cell types, plots for samples 18-0282 and 19-0142, logFC of gene expression in a) palisading necrosis niche compared to the generic tumor region, and b) perivascular niche compared to the generic tumor region. Whether the logFC of the gene is significant is shown in the legend. Adjusted p values < 0.05 were labeled as significant. [file 40478_2024_1769_MOESM12_ESM.pdf]

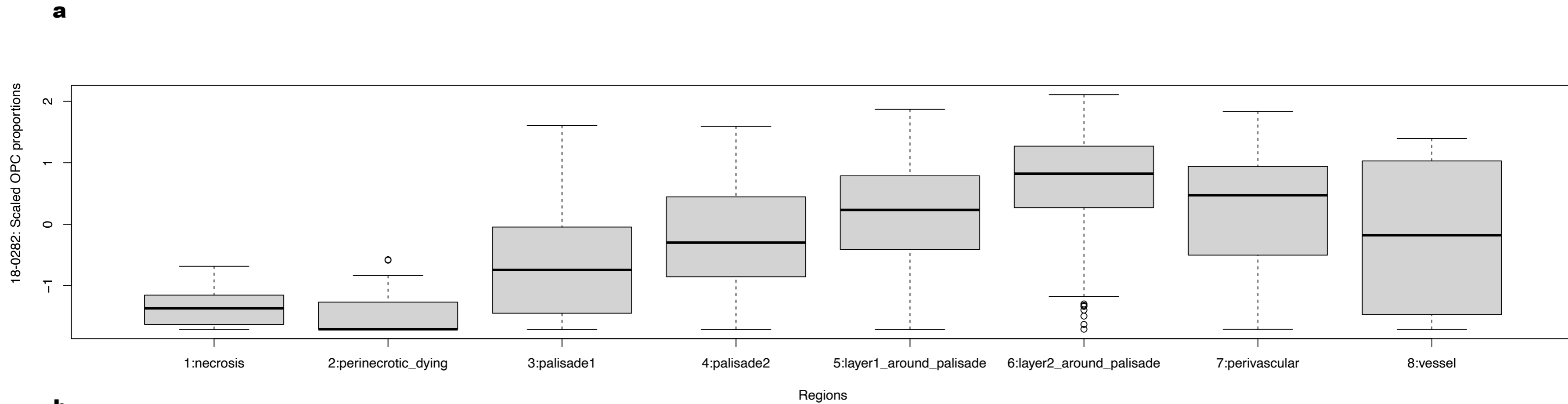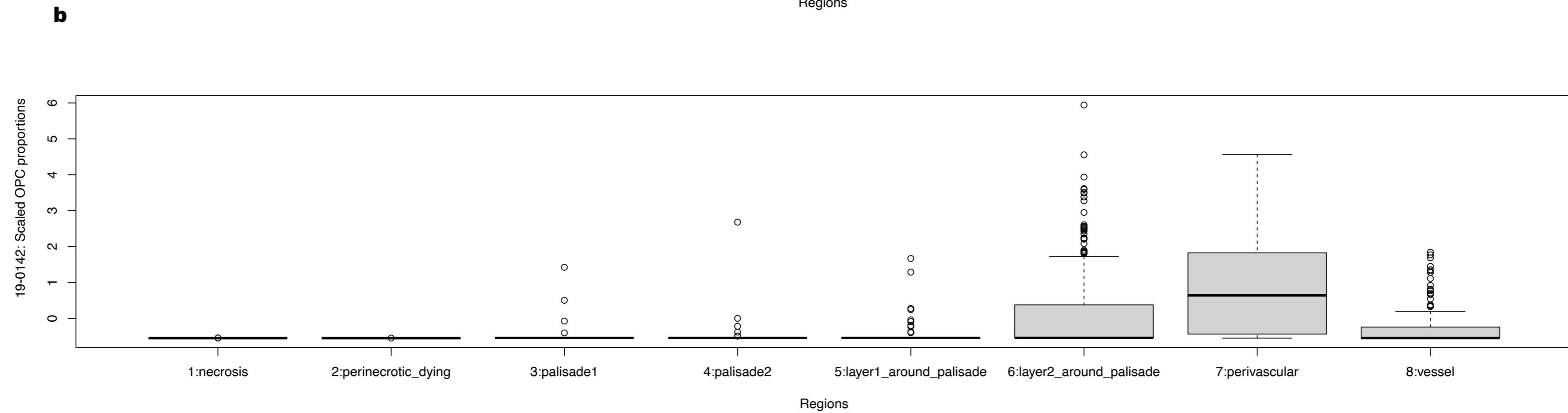

Supplement: Supplementary file 14 — Additional file 14: Fig. S14. Boxplots showing the enrichment of OPC-like tumor in each region, for samples 18-0282 and 19-0142 respectively. [file 40478_2024_1769_MOESM14_ESM.pdf]

Activation z-score 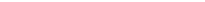 -9.291 8.222

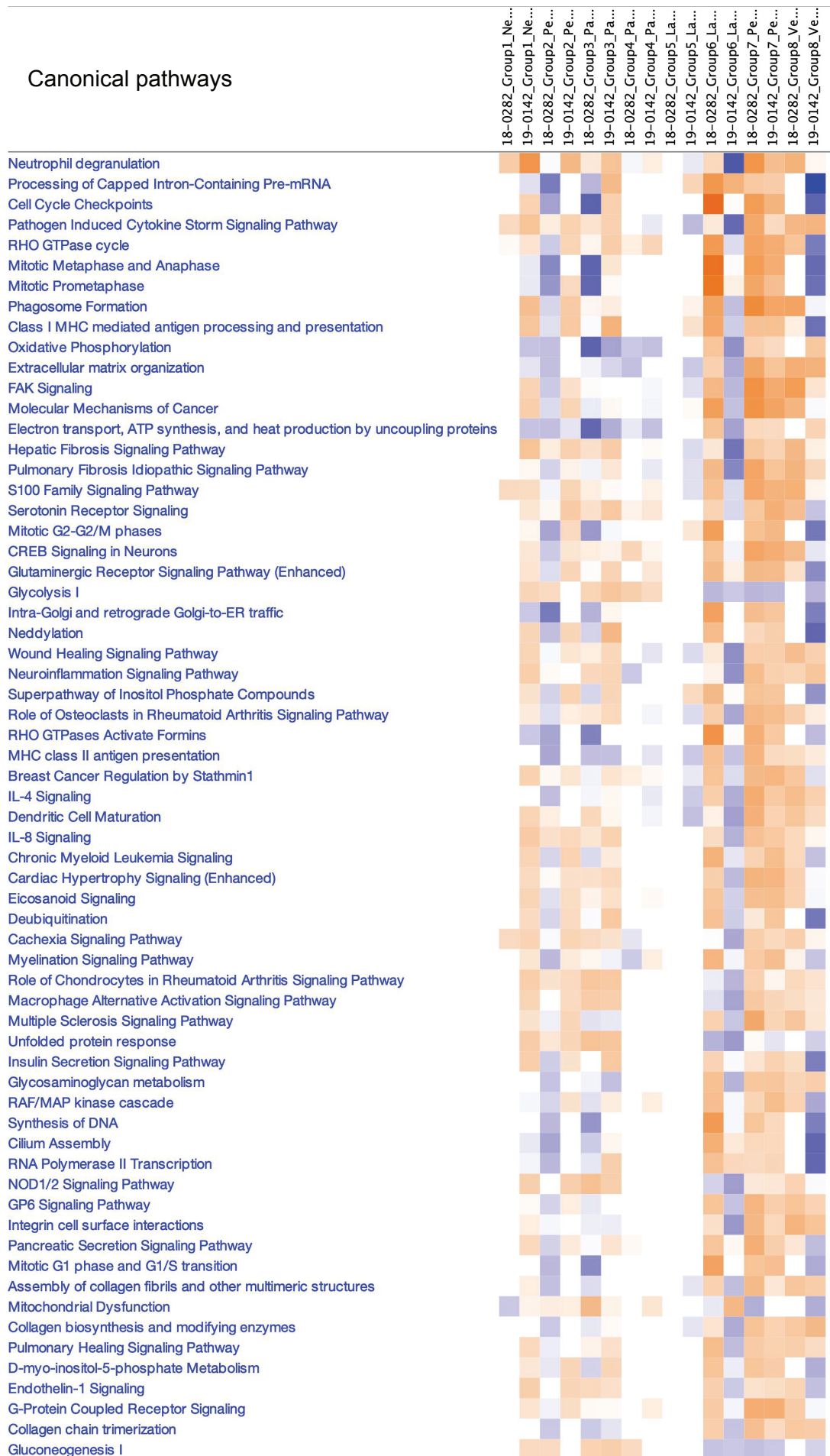

Supplement: Supplementary file 16 — Additional file 16: Fig. S16. Significant pathways in each annotated region, adjusted for cell types, revealed by Ingenuity Pathway Analysis (partial results), FDR < 0.05. Warmer/cooler colors are up/downregulated pathways. [file 40478_2024_1769_MOESM16_ESM.pdf]

Both Not Adjusted for Cell Type Proportions

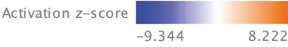

Canonical pathways

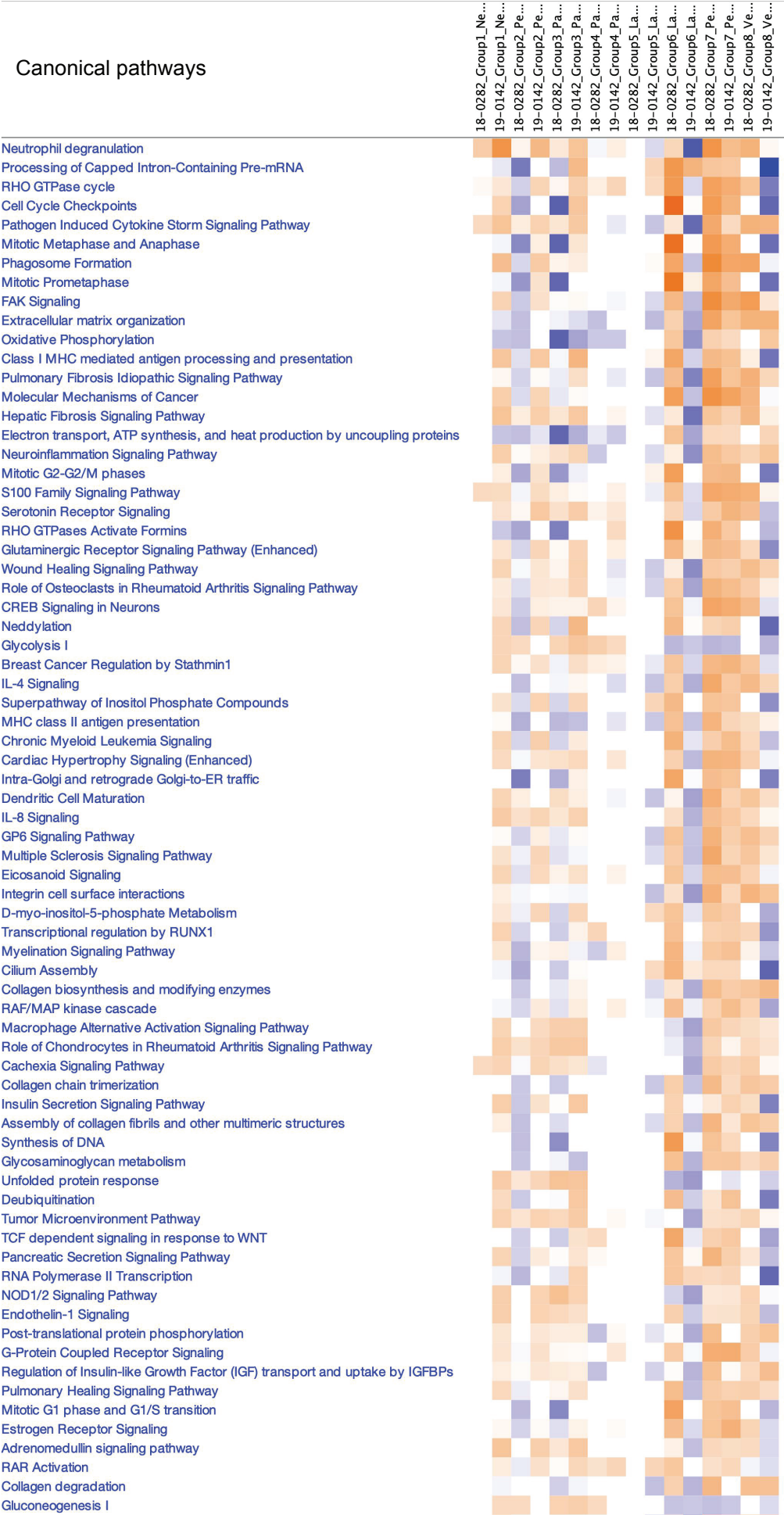

Supplement: Supplementary file 17 — Additional file 17: Fig. S17. Significant pathways in each annotated region, not adjusted for cell types, revealed by Ingenuity Pathway Analysis (partial results), FDR < 0.05. Warmer/cooler colors are up/downregulated pathways. [file 40478_2024_1769_MOESM17_ESM.pdf]
