# Supplementary material for: Spatial transcriptomics reveals segregation of tumor cell states in glioblastoma and marked immunosuppression within the perinecrotic niche
Source: Acta Neuropathol Commun. 2024 Apr 22;12:64. doi: 10.1186/s40478-024-01769-0 (PMC11036705; doi:10.1186/s40478-024-01769-0)
Supplement: Supplementary file 3 — Additional file 3: Fig. S3. Annotation of the gradient of regions for samples 18-0282 and 19-0142, respectively. [file 40478_2024_1769_MOESM3_ESM.pdf]

a b

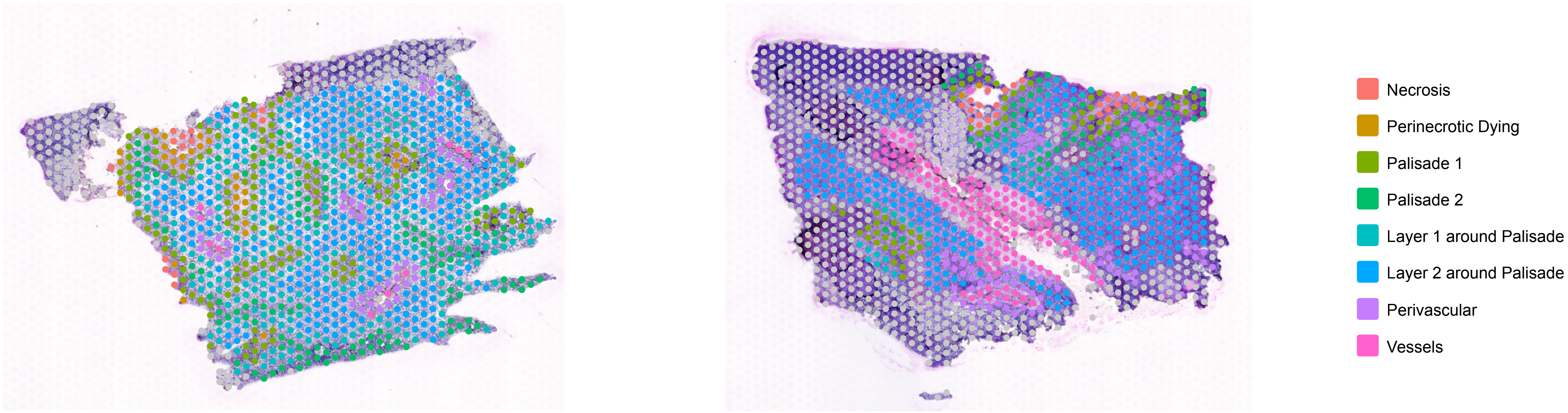
Necrosis
Perinecrotic Dying
Palisade 1
Palisade 2
Layer 1 around Palisade
Layer 2 around Palisade
Perivascular
Vessels
