# Supplementary material for: Spatial transcriptomics reveals segregation of tumor cell states in glioblastoma and marked immunosuppression within the perinecrotic niche
Source: Acta Neuropathol Commun. 2024 Apr 22;12:64. doi: 10.1186/s40478-024-01769-0 (PMC11036705; doi:10.1186/s40478-024-01769-0)
Supplement: Supplementary file 11 — Additional file 11: Fig. S11. Plot for samples 18-0282 and 19-0142, logFC of gene expression in perivascular niche compared to the generic tumor region, cell type adjusted. Whether the logFC of the gene is significant is shown in the legend. Adjusted p values < 0.05 were labeled as significant. [file 40478_2024_1769_MOESM11_ESM.pdf]

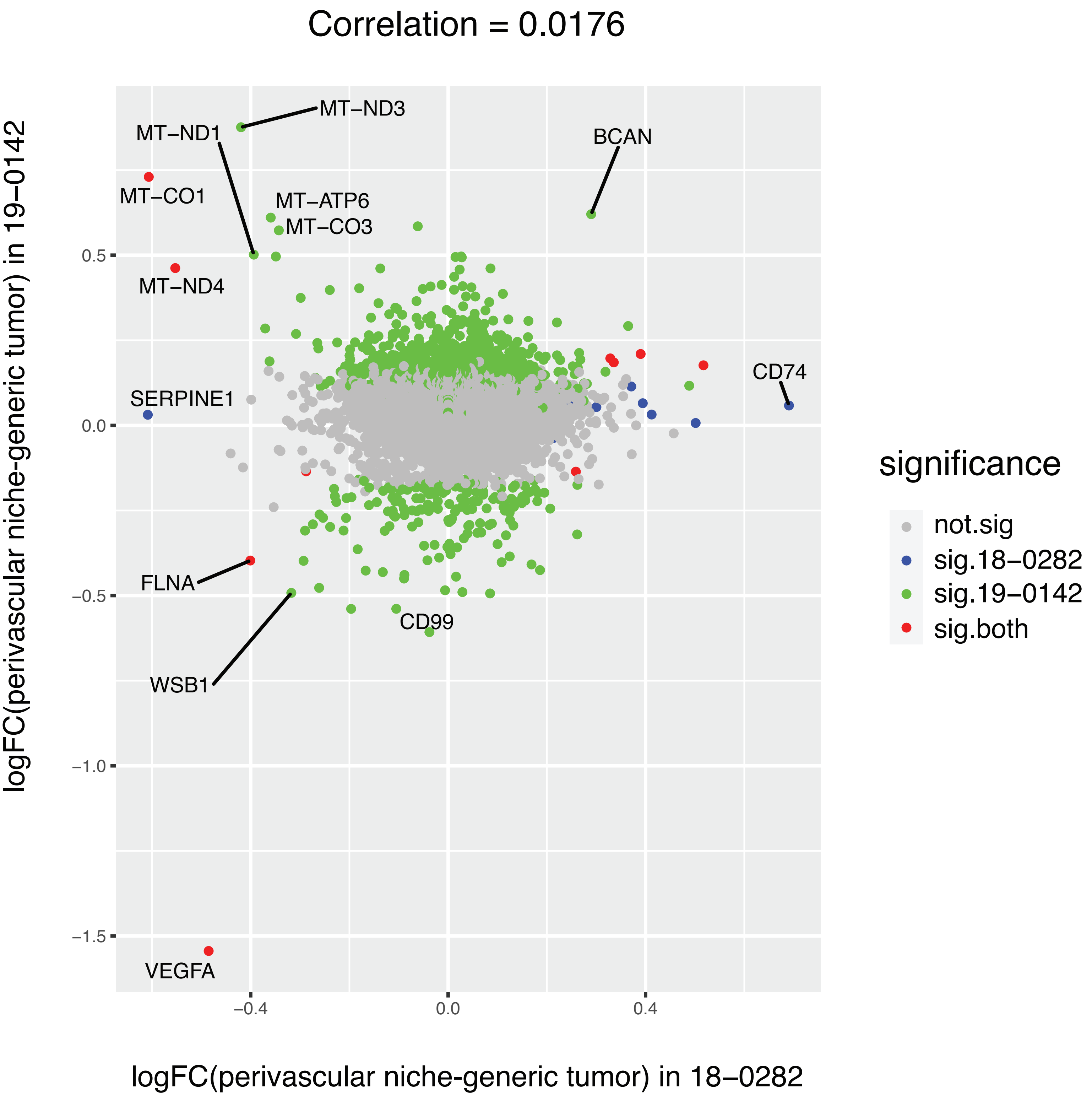

Correlation = 0.0176
logFC(perivascular niche-generic tumor) in 19−0142
logFC(perivascular niche-generic tumor) in 18−0282
MT−ND3
MT−ND1
BCAN
MT−CO1
MT−ATP6
MT−CO3
MT−ND4
CD74
SERPINE1
FLNA
WSB1
CD99
VEGFA
0.5
0.0
−0.5
−1.0
−1.5
−0.4
0.0
0.4
significance
not.sig
sig.18−0282
sig.19−0142
sig.both
